# Bouncing back from COVID-19: a Western Australian community perspective

**DOI:** 10.3389/fpubh.2023.1216027

**Published:** 2023-08-04

**Authors:** Kiira Karoliina Sarasjärvi, Paola Chivers, Ranila Bhoyroo, Jim Codde

**Affiliations:** ^1^Institute for Health Research, The University of Notre Dame Australia, Fremantle, WA, Australia; ^2^Research Program Unit, Faculty of Medicine, University of Helsinki, Helsinki, Finland; ^3^School of Medical and Health Sciences, Edith Cowan University, Joondalup, WA, Australia

**Keywords:** COVID-19, latent transition analysis, behavioral change, health promotion, health policy, resilience, mental health, survey research methodology

## Abstract

**Introduction:**

This study explored the behavioral profiles of residing Western Australians during a COVID-19 lockdown period and transitions in behavior post-lockdown.

**Methods:**

A total of 313 participants (76% female, age: M = 50.1, SD = 15.7 years) completed behavioral and mental health questionnaire items ~2 months after a 3-month COVID-19 lockdown in October 2020, using a retrospective recall to assess their experience during the lockdown period. Latent transition analysis (LTA) was used to identify behavioral profiles and transitions. Indicators were identified by assessing during–post-lockdown group differences (Kruskal–Wallis, chi-square tests) and profiles described using qualitative open-ended questions.

**Results:**

Significant indicators included changes in physical activity, leisure screen time, alcohol intake, psychological distress, and loneliness, but not fast food consumption. The significant indicators were used to form LTA models. The five latent class model showed the best model fit (Log-likelihood = −1301.66, AIC = 426.12, BIC = 609.68). Approximately one in four participants reported a change in their behavior profiles after the lockdown ceased. Key differences between the profiles were age, household income, education, resilience, sense of control, existing mental health issues, and social relations. Washing hands and social distancing were the most recalled and effective health campaigns across the classes, with health campaigns encompassing physical activity/alcohol consumption, or domestic violence having the least attention.

**Discussion:**

Overall, while most participants recovered relatively well after the lockdown period, LTA did identify subgroups such as those who were inactive and lonely experienced more difficulties than other groups, and engagement with public health campaigns differed. The results provide important insights for future public health campaigns on how these campaigns might be diversified to effectively target more people and particular groups to maximize engagement for maintaining people's mental health with additional focus on physical activity, alcohol consumption, and domestic violence.

## 1. Introduction

The COVID-19 pandemic continues to affect communities across the world in different ways. Globally, mental health deteriorated in early 2020, when countries across the world were implementing COVID-19 restrictions (e.g., lockdowns) to stop or slow virus transmission and infections. A meta-analysis encompassing 65 longitudinal studies, predominantly from Europe and North America, reported small but significant increases in mental health-related symptoms among the general population and for people with pre-existing physical health conditions (1). The increase in mental health symptoms in relation to pre-pandemic levels was higher in the early stages of the pandemic (March–April 2020) but has been reported to have almost returned to pre-pandemic levels in May–July 2020 (1).

Countries such as Australia that imposed hard lockdown procedures (e.g., movement restrictions, curfews, and school closures) were in a unique position compared to other countries by having low infection and death rates at the start of the pandemic (2). A report from the Australian Institute of Health and Welfare (AIHW) examined the indirect health effects of COVID-19 (2). Similar to the meta-analysis (1), this report (2) also identified an increase in psychological distress during the peak of the pandemic that reverted to pre-pandemic levels by April 2021, except for younger people aged 18–44 years and those experiencing severe psychological distress. Similar results were also reported for loneliness, with almost half of participants reporting feeling lonely for at least some of the time in April 2020 but the level dropping to ~10% by the end of May (2).

However, there is growing evidence that not everyone was affected the same way during the pandemic, with some people improving, some worsening, but most reporting no change in their behavior during the start of the pandemic. For example, individual differences in behavioral changes (e.g., physical activity, fast food consumption, alcohol intake, and screen use) during the pandemic were observed in the AIHW report (2). Investigating these changes at a group level during COVID-19 lockdowns can provide important insights for developing targeted health campaigns for specific cohorts. Two studies (3, 4) examined the different health profiles (e.g., mental health and substance use) in tertiary students before and during COVID-19 by using latent class analysis (LCA) and latent transition analysis (LTA). LCA is a statistical technique that helps establish unknown subgroups within a population, whereas LTA is the longitudinal version of LCA that allows individuals to transit between the groups (5, 6). For example, the USA study that examined student behavior found that mental health problems increased before and during COVID-19 lockdowns, whereas substance use, sexual behavior, physical inactivity, and food insecurity decreased during COVID-19 lockdowns (4). Similarly, a German study revealed five behavioral classes encompassing substance use (3). Students who occasionally used different substances seemed to change their behavior during the pandemic, with most of them stopping substance use, whereas non-consumers or regular smokers did not change their behavior (3).

Furthermore, two studies from Australia examined subjective change in people's behavior (e.g., physical activity) during the lockdown period in relation to both before (7) and after (8) the lockdown by using retrospective recall. For example, a study from Queensland reported a significant association between negative change in behavior (physical activity, sleep quality, smoking, and alcohol intake) and mental health symptoms (depression, anxiety, and stress) before and during the lockdown (7). Furthermore, the study by Bhoyroo et al. (8) revealed similar polarizing effects of COVID-19 during and 2 months after the initial lockdown in Western Australia in 2020. Similar to the AIHW report (2), these results suggest that while some individuals struggled to adapt to the lockdown, others indicated an improvement in their health during the lockdown period, while the majority of participants reported no change in their behavior during the lockdown period.

Another important, but far less studied, research area is the impact of health promotion campaigns and the public's health literacy skills during the time of COVID-19. According to the World Health Organization (WHO), health promotion is a process that enables individuals to improve and to be more in control over their own health (9). Health literacy, on the other hand, is often defined as individuals' ability to find, understand, and use health-related information to guide their decisions and actions regarding their health (10). Both are important skills for successful public health communication. During COVID-19, the number of health promotion campaigns, especially in health-related (e.g., hand washing, hygiene, exercise, and mental health), was increased in Australia (11) and around the world (12–15). While some studies examined the cost-effectiveness of government actions during the pandemic (16, 17) or explored the impact of strategies and policies implemented in different countries (16, 18, 19), or mitigation versus containment policies within a country (20) on the spread of COVID-19, very few studies have investigated the relationship between health promotion campaign recall and the change in people's behavior during the COVID-19 pandemic (8, 21). For example, Bhoyroo et al. (8) investigated how well residents of Western Australia were able to recall different health campaigns during the COVID-19 pandemic. Most commonly recalled health promotion messaging was related to washing hands or social distancing, whereas health promotion campaigns encompassing physical activity, nutrition, and mental wellbeing were less recalled but identified as required by respondents (8). Another study from the US reported a positive relationship between increased vaccine confidence and the participant's ability to recall health promotion campaigns, emphasizing the importance of memorizing the health promotion campaigns to change people's behavior (21).

The present study extends the study by Bhoyroo et al. (8) that explored behavioral changes (physical activity, diet, alcohol intake, and mental health symptoms) during and after the COVID-19 lockdown among the general population in Western Australia. However, far less is known about how these behavioral changes are linked to each other, or how people recovered from COVID-19 lockdowns. The present study aimed to examine these differences using an LTA approach to form behavioral profiles and investigate the transitions between these LTA profiles. Open-ended responses were used to enhance the understanding of these behavioral changes related to different LTA profiles. The objectives of this study were as follows:

1. *To use an LTA approach to form and explore the behavioral and mental health profiles during and after the COVID-19 lockdown*.
2. *Investigate the groups differences and transitions between the LTA profiles, when returning to “normal life” after the COVID-19 lockdown*.

## 2. Materials and methods

This study was based on a survey research methodology design and reports on a subset of participants (*n* = 313) who completed all questions in the Health and Wellbeing study (*n* = 547) by Bhoyroo et al. (8) and therefore followed the same study procedures. Ethics approval was obtained from the institution's Human Research Ethics Committee (REF 2020-133F). Western Australian residents (aged 18 years and above) completed a cross-sectional online survey using Qualtrics (22), ~2 months after the lockdown (from mid-August to October 2020). During this single survey, participants were asked to retrospectively recall and assess their behavior during the COVID-19 lockdown (2 months earlier) and at present (after COVID-19 lockdown restrictions had been lifted). The priori sample size calculation using G^*^Power (23) considering the group and timepoint comparisons (medium effect size = 0.31, α = 0.05, power = 0.80) yielded a minimum of 174 respondents. For a more detailed description of the study procedures, see Bhoyroo et al. (8).

The study by Bhoyroo et al. (8) revealed several individual changes in behavior and mental health during and after the COVID-19 lockdown. Similarly, drawing from the same sample as Bhoyroo et al. (8), Piggott et al. (24) more deeply investigated the association between physical activity, sedentary behavior, and mental health symptoms during the COVID-19 lockdown. Since the LTA offers a unique approach to explore the hidden subgroups that the previous conventional methodological approach (e.g., regression, within-subject analysis, or group comparisons) cannot detect, the present study expands the earlier research by Bhoyroo et al. (8) and Piggott et al. (24) by focusing on group behavior rather than individual- or population-level changes.

### 2.1. COVID-19 pandemic and lockdown procedures in Western Australia March–June 2020

The state of Western Australia underwent an initial lockdown from 23 March 2020 for ~3 months that involved restriction of residents' movements restricted (e.g., limited outdoor exercise and intra-state travel), closure of social venues (e.g., gyms, theaters, and dining in restaurants), and work and education from home, except for essential workers (e.g., hospital, police, and emergency service) (25–28). Some of the restrictions started to ease from mid-May until early June (25). During this period, the direct impact of COVID-19 remained low, with the total number of confirmed cases by 27 June 2020 being 599 with daily cases ranging between 0 and 34. By the end of the data collection (21 October 2020), the cumulative confirmed cases remained relatively low at 752 cases, resulting in the level of state-based restrictions being gradually eased (29) and the opening of interstate and overseas borders 2 years later on 3 March 2022 (30).

### 2.2. Measures

#### 2.2.1. LTA model primary measures

The LTA was chosen as a statistical approach since it is suitable when the aim was to investigate the ways in which individuals transit between subgroups over time periods. All the tested models had two timepoints (during and after the COVID-19 lockdown), which were assessed by using a retrospective recall. Physical activity, screen time, fast food consumption, alcohol intake, psychological distress, and loneliness were assessed by using Wilcoxon's signed rank tests and later by LTA. All indicators were measured twice: during lockdown (retrospective recall) and after lockdown (time of data collection). All questions, except those regarding loneliness, were adopted from the Western Australia Health and Wellbeing Surveillance System (WA-HWSS) survey (31).

##### 2.2.1.1. Physical activity

Physical activity levels *(‘How would you rate your physical activity level?')* were assessed using a 5-point Likert scale, with higher numbers indicating higher activity levels. The variable was dichotomized (inactive, active), where the two lowest values were coded as “inactive” (1 = not active at all and 2 = not very active) and all the other values were coded as active (3 = moderately active, 4 = active, and 5 = very active).

##### 2.2.1.2. Leisure screen time

Leisure screen time was assessed by asking participants to report how many hours per week (excluding work time) they spend watching or using different electronic devices. Due to the lack of screen time recommendations for adults, the cutoff was set based on Western Australian leisure screen time recommendations (maximum 2 h/day) for children and young people (32). The cutoff was set to 14 h/week, which will be later referred as “less” and “more than 2 h a day.”

##### 2.2.1.3. Fast food consumption

Fast food consumption was reported by asking how many times per week participants eat fast food meals or snacks (e.g., McDonalds). The variable was dichotomized to “not at all” and “at least once a week.”

##### 2.2.1.4. Alcohol intake

Alcohol intake was assessed by asking, “*On a day when you drink alcohol, how many standard drinks do you usually have?*”. The cutoff point was set to 4 standard drinks aligned with the healthy recommendations given by the Australian Government Department of Health (33). Responses were dichotomized to “less” and “more than 4 drinks a day.”

##### 2.2.1.5. Mental distress (K-6)

In contrast to the 10-item Kessler psychological distress scale (K10) utilized in the study by Bhoyroo et al. (8), the present study utilized the shorter 6-item version (K6). The K-6 version was chosen due to the validated “dichotomic” categorization: “No probable serious mental illness” (sum range 6–18) and “Probable serious mental illness” (sum range 19–30) established by the Australian Bureau of Statistics (34). The K-6 includes six items from the original K-10 version: *felt nervous, hopeless, restless or fidgety, worthless, everything was an effort*, and *so sad/depressed that nothing could cheer you up*, and it has been validated among the Australian general population (35, 36). The internal consistency (Cronbach's alpha) in the present study was 0.90 and 0.91 during and after the COVID-19 lockdown, respectively.

##### 2.2.1.6. Loneliness (UCLA-3)

The three-item loneliness scale (37) was adopted from the R-UCLA Loneliness Scale (38). The sum scored ranged between 3 and 9, with higher scores indicating higher levels of loneliness. The sum scores were transformed into a dichotomous variable (not lonely ≤ 3, lonely >3). The internal consistency in the present study was 0.85 and 0.84 during and after the COVID-19 lockdown, respectively, which is slightly better than the internal consistency of the original study by Hughes et al. (37) (α < 070).

#### 2.2.2. LTA model secondary measures

After forming the LTA classes, the group differences were investigated by using scale score variables. These variables included age (years), resilience [Brief Resilience Scale, BRS, (39)], number of social groups (e.g., church, sporting, political, and professional groups) before COVID-19, family functioning (4 items), and lack of control (3 items). Categorical variables included sex (male, female), country of origin (Australia, outside of Australia), identifying as indigenous (yes, no), highest education (university degree, lower education), household income ($100,000 or more, <$100,000), currently receiving treatment for mental health issues (yes, no) and the starting date of the treatment (before, during, after lockdown), subjective experience of change in family functioning (better than normal, about the same, worse than normal), and involvement in social groups during the lockdown (less, about the same, more).

##### 2.2.2.1. Resilience (BRS)

Resilience is defined as an ability to bounce back or recover from stress or stressful event (39). It is important that this term is not confused with resisting or adapting to stress, or thriving from stress (39). Resilience was assessed after lockdown using 6-item Brief Resilience Scale (BRS) (39). Higher average indicates higher resilience (1 = strongly disagree, 5 = strongly agree), with three of the items (2, 4, 6) using reversed scaling. The scale has been proven to follow a single-factor structure with high internal consistency (α > 0.80) (39, 40). In Australia, a single-factor model accounting for negative items showed an excellent fit (40). The internal consistency (Cronbach's alpha) in the present sample was 0.89.

##### 2.2.2.2. Family functioning

Family functioning was calculated by averaging 4 items regarding family functioning: “*in general, my family don't get well-together,” “planning family activities is usually difficult,” “usually avoid discussing our fears and concerns openly with each other,” “making decisions is usually a problem in our family because we misunderstand each other*.” The mean scale ranged between 0 and 3, where higher values indicated higher family functioning (3 = strongly disagree, 0 = strongly agree).

##### 2.2.2.3. Lack of control

To assess lack of control during and after lockdown, the mean scores (range 0–4) were calculated for both timepoints combining 3 items measuring the lack of control i*n general, in personal life*, and *in health*. Higher average indicated higher sense of control (0 = Never, 4 = Always).

### 2.3. Statistical analysis

Preliminary analyses of the demographic and behavioral measures of the initial sample (8) (*n* = 547) and the final sample utilized in this study (*n* = 313) were compared with chi-square, Mann–Whitney U-tests, and Dunn's *post-hoc* comparisons.

Wilcoxon's signed rank tests of the primary measures (physical activity, leisure screen time, fast food consumption, alcohol intake, mental distress, and loneliness) were used to determine the significant indicators to form LTA.

LTA was conducted to generate and test several models with different numbers of classes to decide the most optimal number of classes based on the data (5). In the present study, the primary measures contained two timepoints (during and after lockdown) which were assessed by using a retrospective recall. To form the LTA, only the statistically significant primary measures were considered, and the LTA identified two to seven classes. The best LTA model fit was assessed based on the lowest possible likelihood-ratio G^2^ statistic, Akaike's information criterion [AIC; (41)], and Bayesian information criterion [BIC; (42)] along with the interpretability of the models (6). For example, the characteristics of each class were required to be distinct, and small class sizes, <5%, were excluded.

Once the most optimal number of classes was established, we compared the groups that either remain in their class or transit between the classes after lockdown ceased. The group differences were assessed based on the secondary measures (e.g., socio-demographic variables, resilience, family functioning, and lack of control) to seek the possible socio-demographic differences between the groups. Kruskal–Wallis test, Dunn's *post-hoc* comparisons, and chi-square test were used to test these differences.

IBM SPSS version 27 (43) was used to describe variables and conduct all the group comparisons. The LTAs were carried out by using publicly available PROC LTA (44) developed for SAS Version 9.1 for Windows (45). Alpha < 0.05 (two-sided) was considered statistically significant.

#### 2.3.1. Open-ended questions

To gain a better understanding of the behavioral changes and challenges between the LTA classes, several open-ended questions were assessed (Table 1). Thematic analysis was used to assess the open-ended questions regarding physical activity, screen time, diet, alcohol consumption, and mental health. The method was used to enhance the understanding of the characteristics of specific latent classes obtained from LTA using primary measures, whereas questions encompassing social network and family relation were assessed to deepen the understanding regarding the group differences based on the secondary measures.

**Table 1:** Open-ended questions.

	**Topic**	**Open-ended question**
Characteristics of the LTA groups	Physical activity	Thinking back to COVID-19 lockdown, what would you say was the biggest difference made to your physical activity (in other words, any changes to your physical activity preferences, types of physical activity, physical activity intensity, etc.)? Please describe those changes
		Are there any other comments you would like to make about any changes to your physical activity levels, habits and choices? Please explain what has changed and why you think it has changed during this time
	Diet and alcohol intake	Thinking back to COVID-19 lockdown, what would you say had been the biggest difference you made to your diet (in other words, any changes to your food preferences, types of food, food preparation, cooking, alcohol intake etc.)? Please describe what changed
	Mental health	Describe what may have affected (positively and/or negatively) your mental wellbeing during the COVID-19 lockdown period?
Comparisons between LTA groups	Sense of control	Describe what may have affected (positively and/or negatively) your feelings of control during the COVID-19 lockdown period?
	Effects of COVID-19	Are there any other comments you would like to make about the impact of COVID-19 on physical and mental wellbeing?
		Provide examples of how you and your family were positively affected
		Provide examples of how you and your family were negatively affected
	Health promotion	Please list the topic of any of the health promotion campaigns you recall that run during the COVID-19 lockdown period.
		Please describe any changes you made to your behaviors as a result of these campaigns
		What health promotion messages should have been provided to the community during COVID-19 lockdown that were missing at the time?

Content analysis was used to assess the open-ended questions regarding the ability to recall existing health promotion campaigns and assess the behavioral changes due to these campaigns. The responses were extracted up to three unique points from each respondent. The exact wording of the extracted points was themed and assigned a numerical code to enable frequency analysis based on the multiple responses to each question (46).

## 3. Results

The pre-analysis revealed a large number of records of systematic missing values. In our subsample, participants who had more than 50% values missing were excluded, yielding a total of 313 responses.

Comparative analysis revealed that the subsample used in this study (*n* = 313) was statistically similar to the original sample (8) across all of the dimensions measured (Supplementary Table S1). Of note, those who were excluded from this study were younger [*t* (467.63) = −3.45, *p* < 0.001], more likely to be born in Australia [χ2(1) = 6.038, p = 0.014], and consumed more fast food [χ2(1) = 8.125, *p* = 0.004] and alcohol [χ2(1) = 5.106, p = 0.024] after lockdown finished (Supplementary Table S1).

Most respondents in this study were female (76.1%) and middle-aged (M = 50.1 years, SD = 15.7 years) and had a university degree (69.7%). Approximately half of the participants had an annual household income of at least AU$100,000 (50.6%) or were born in Australia (61.0%). Less than two percent identified themselves as indigenous.

The behavioral characteristics during and after the initial lockdown period are presented in Table 2. People reported being significantly more inactive, more time spent in screen time activity, drank more alcohol, had more severe mental health symptoms, and felt lonelier during the lockdown. There was no significant change in eating fast food (*p* = 0.668) with ~40% consuming fast food at least once a week at both timepoints. Since no significant change was detected, fast food consumption was not included as a variable in the LTA models.

**Table 2:** Behavioral changes during and after lockdown (*N* = 313).

**Indicators**	**Behavioral changes**	**During lockdown % (*n*)**	**After lockdown % (*n*)**	**Wilcoxon**	***p*-value**
Physical activity	Inactive	34.7 (107)	20.1 (62)	−5.063	*p* < 0.001
	Active	65.3 (291)	79.9 (247)
Leisure screen time	Less than 2 h/day	53.4 (163)	66.2 (202)	−5.689	*p* < 0.001
	More than 2 h/day	46.6 (142)	33.8 (103)
Fast food consumption	Not at all	60.5 (184)	59.5 (181)	−0.429	*p* = 0.668
	At least once a week	39.5 (120)	40.5 (123)
Alcoholintake	Less than 4 drinks	91.5 (270)	95.6 (281)	−3.000	*p* = 0.003
	More than 4 drinks a day	8.5 (25)	4.4 (13)
Psychologicaldistress	No probable	87.7 (264)	91.5 (279)	−2.121	*p* = 0.034
	Probable serious mental illness	12.3 (37)	8.5 (26)
Loneliness	Not lonely	30.8 (91)	45.2 (135)	−4.950	*p* < 0.001
	Lonely	69.2 (204)	54.8 (164)

Variables: Physical activity (inactive; active); screen time (less; more than 2 h/day); alcohol intake (less; more than 4 standard drinks a day); psychological distress (no probable of serious mental illness; probably of serious mental illness); loneliness (no, yes).

### 3.1. Latent transition analysis (LTA)

A total of six models with two to seven latent classes were compared. The model fits are presented in Table 3. Models with classes with < 5% of participants were not included in the final decision. The model with five classes yielded the best fit, having the lowest BIC and second lowest AIC values. More detailed descriptions of each model are presented in Supplementary Tables S2–S7.

**Table 3:** LTA models—fit statistics.

**Fit statistics**	**2 classes**	**3 classes**	**4 classes**	**5 classes**
Log-likelihood	−1426.57	−1389.30	−1377.59	−1301.66
G-squared	577.96	503.42	479.99	328.12
AIC	603.96	549.42	549.99	426.12
BIC	652.66	635.58	681.10	609.68
Degrees of freedom	1,010	1,000	988	974

Best model in highlighted in gray.

**Figure 1:**
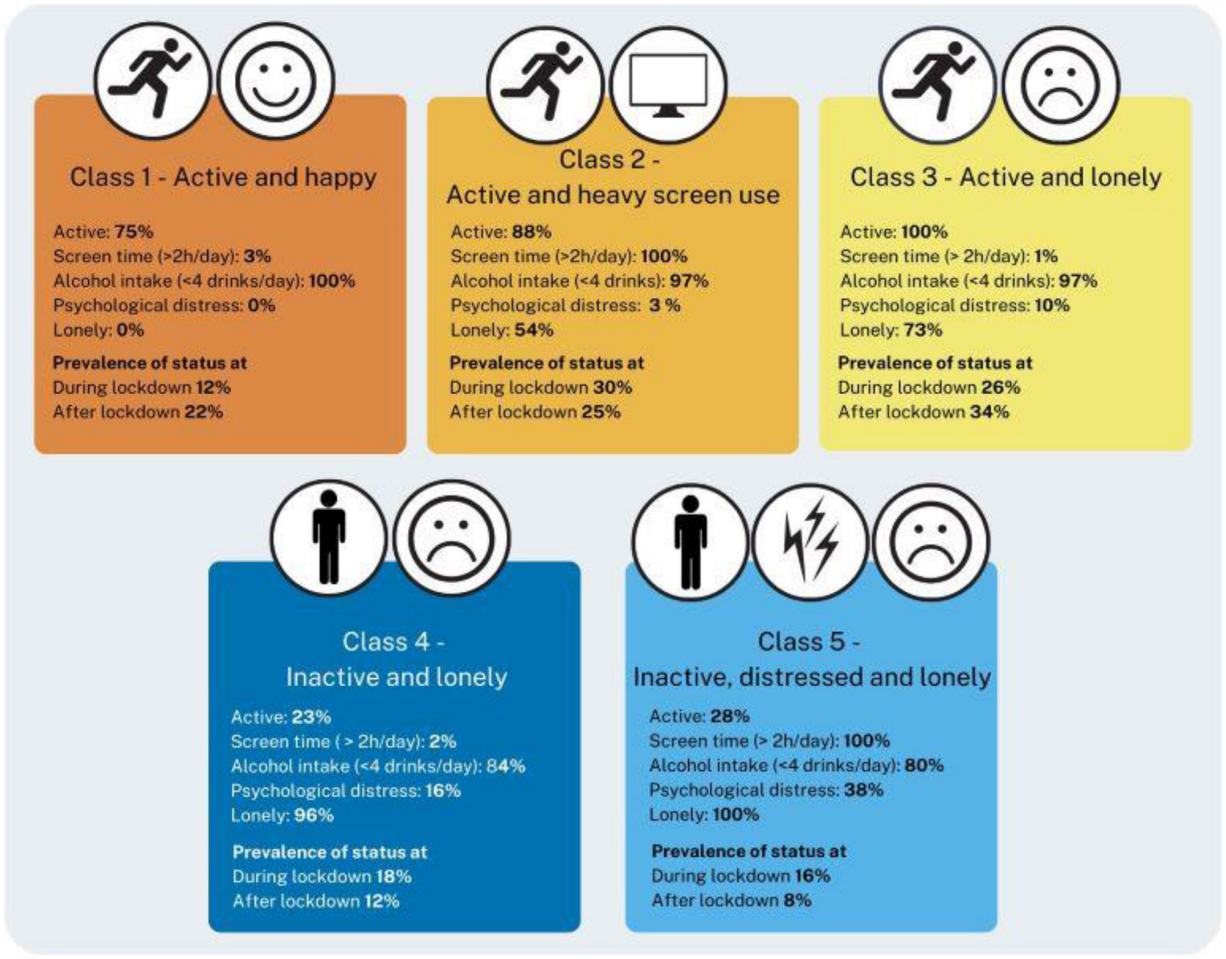
LTA model with five latent classes: characteristics and prevalence of status during and after lockdown.

The five latent classes differed mainly on physical activity levels, leisure screen time use, and loneliness. Alcohol intake and psychological distress showed only minor differences between the classes, with inactive groups drinking more alcohol and being more distressed. As a consequence, the five classes were named as follows: *Class 1) Active and Happy, Class 2) Active and Heavy Screen Use, Class 3) Active and Lonely, Class 4) Inactive and Lonely, Class 5) Inactive, Distressed and Lonely*. The sizes of the five latent classes ranged between ~10% and ~30%. During the lockdown, almost a third (30%) of participants belonged to *Active and Heavy Screen Use*, a quarter (26%) *Active and Lonely*, and one-fifth (18%) to *Inactive and Lonely*. Approximately 16% belonged to *Inactive, Distressed and Lonely*, whereas only 12% were *Active and Happy*. After lockdown, *Active and Lonely* had the highest prevalence of 34%, followed by *Active and Heavy Screen Use* (25%) and *Active and Happy* (22%). Approximately every tenth belonged to either *Inactive and lonely* (12%) or *Inactive, Distressed and Lonely* (8%). Note that Figure 1 only illustrates the characteristics and prevalence of each class in different timepoint but does not show the individual transitions between these classes between during and after COVID-19 lockdown periods.

#### 3.1.1. Transition between LTA classes

**Figure 2:**
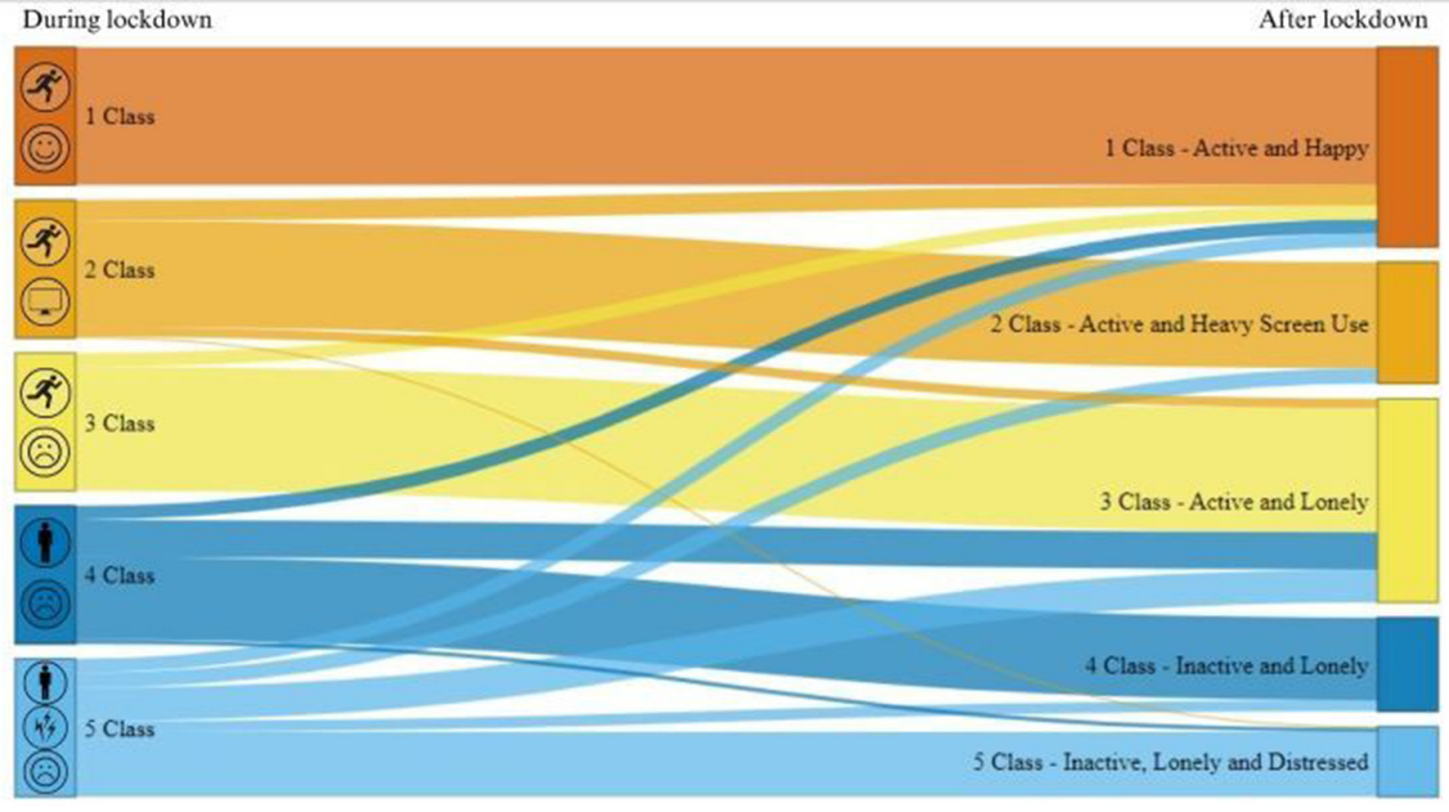
Prevalence and transitions between five latent classes during and after COVID-19 lockdown.

The prevalence and transitions between the classes during and after lockdown are presented in Figure 2.

People who were already active during the lockdown period mostly remain in their class (77–100%) after lockdown ended in comparison with those who were inactive (47–60%). However, approximately a quarter (24.6%, *n* = 77) of all the participants transit from their initial class (during the lockdown) to another after lockdown ceased. Within this group, most people were able to bounce back after lockdown, whereas some individuals struggled after lockdown and their health and wellbeing outlook got worse after lockdown. The more detailed descriptions of these groups are discussed below. The numeric values of the characteristics and transitions are displayed in Supplementary Table S2.

##### 3.1.1.1. Transitions (*n* = 77)

Despite marked variation between the classes, almost 25% (24.6%, *n* = 77) of participants who transitioned from their initial class to another after lockdown ceased with the majority (88.3%, *n* = 68) moving to a more positive group (e.g., *Active and Happy*). However, 11.2% (*n* = 9) transitioned to a more negative health and wellbeing outlook, such as *Active and Lonely* or *Inactive, Distressed and Lonely* (Figure 2).

Approximately 10% of people from each group transit into *Active and Happy* group after lockdown suggesting that most people were able to “bounce back” after lockdown finished. Furthermore, approximately a quarter of people initially in an “inactive” group shifted into *Active and Lonely*. Even though these people became more active and less distressed, they remain lonely. Additionally, approximately 10% of the *Inactive, Distressed and Lonely* group transit to either *Active and Heavy Screen Use* or *Inactive and Lonely* groups after lockdown, but these subgroups differed greatly in their behavior. People who shift to *Active and Heavy Screen Use* became more active, did not change their screen time, but were less lonely and distressed. In contrast, people, who transit to *Inactive and Lonely*, remain inactive and lonely but spent less time on their screen and were less distressed.

Alarmingly, the results revealed some people struggled to recover from lockdown with a small portion of people from *Active and Heavy Screen Use* (1%) and *Inactive and Lonely* (3%) transitioning to the Inactive, Distressed and Lonely group after lockdown. These people seem to in general feel worse after lockdown than before, becoming lonelier and more distressed. In addition, 7% of people from *Active and Heavy Screen Use* group shifted to *Active and Lonely* after lockdown, reporting they felt lonelier and more distressed, and reduced their leisure screen time usage.

##### 3.1.1.2. Class 1: remain active and happy (*n* = 38)

Everyone classed as *Active and Happy* during the lockdown period remained *Active and Happy* after lockdown ended, suggesting that lockdown had no or minimal impact on their behavior. These individuals remained active, their screen use and alcohol intake stayed low, and none of them felt distressed or lonely after lockdown. However, only 12% (*n* = 38) of all the participants belonged to this group.

In their open-ended responses, many people in this group reported positively on how COVID-19 and the lockdown had impacted their life. For example, people mentioned either no change or a decreased intensity in their physical activity; people walked more and spent more time outdoors with their family than usual since they were working from home and had more time in general. Despite the seemingly resilience of this group, many mentioned being worried for others, experiencing work-related stress (i.e., job insecurity), or other concerns regarding the COVID-19 virus in general. A few people mentioned an increase in their drinking habits, despite none of these participants reported having more than four standard drinks a day.

*Positive due to time to read and spend time with my children at beach. Negative due to worries about reduction in work and worry about my husband's job* (Female, 45)

##### 3.1.1.3. Class 2: remain active and heavy screen use (*n* = 72)

Despite the lockdown, three quarters (77%, *n* = 72) of people initially in this class remained in this group through to lifting of restrictions. While people stayed active, they continued to spend a lot of time engaged in screen-based activity. They continued to consume alcohol within the recommendations, and they did not feel distress, although half remained lonely.

Like people who remained *Active and Happy*, this group reported either lower intensity, no change, in their exercise habits, along with having more leisure time to exercise, or spend time with their family. Increased leisure time also allowed people to watch more TV and stay in touch with friends and family. Similar to Class 2, some drank more than usual, despite this group also not exceeding the recommended alcohol daily limit. Similarly, the negative impact of COVID-19 and lockdown involved being worried about or missing friends and family, while some mentioned difficulties to adjust to new work routines. However, many mentioned that they were more aware of their choices during the lockdown in general.

*Worry about my parents. Extra work during lockdown, had to teach online and everything was new and time consuming. Walking was good for mental health during lockdown* (Female, 46)

##### 3.1.1.4. Class 3: remain active and lonely (*n* = 76)

Most (90%, *n* = 76) participants in this class remained in the same classification after lockdown. They remained active and had low screen time use and low alcohol intake. However, the majority felt lonely, and some reported feeling of distress.

Similar to previous classes, many managed to adapt to the new situation and were more aware of the importance of keeping up a routine. Some mentioned that they tried online exercising, whereas fairly few decided to focus on other activities instead.

*Group training in gym not allowed - many more walks outside instead of time at gym. Yoga classes canceled - replaced with online yoga movie subscription. Far more active through gardening to try to make up for sedentariness of working from home* (Female, 52)

However, regarding alcohol consumption or change in diet, this class was divided. Some mentioned that they were drinking less due to lack of social gatherings or having a better diet, whereas some reported that they were drinking alcohol and snacking more than usual. The impact of lockdown on mental health was also mixed. Many mentioned that having more leisure time made them feel more relaxed, whereas for some the lockdown caused enormous amount of distress (e.g., work-related stress, negative news, lack of control, and feelings of loneliness).

*Being with family, daily routine, rest was great during lockdown. My stress levels seem to be more from work related loneliness* (Female, 66)

##### 3.1.1.5. Class 4: remain inactive and lonely (*n* = 24)

Almost two-thirds of people (60%, *n* = 24) remained *Inactive and Lonely* after lockdown. These people remain mostly inactive and lonely and spent < 2 h/day looking on screens. These people also had the second highest (to Class 5) for alcohol consumption and prevalence of mental health challenges. Unlike people who were active, this class struggled to adapt to exercising on their own, and therefore, many reported a reduction in their exercise habits during the lockdown. Many also struggled to return to normal routines after lockdown ended.

*I haven't picked back up the activity that I used to do pre lockdown, I don't go to the pool or go for power walks anymore* (Female, 36)

Most people reported that they were missing time spent with their friends and family or that they were feeling lonely and isolated during the lockdown. Similar to the previous classes, some mentioned increased workload, or other work-related concerns.

*Negatively: unable to meet with friends and family. Unable to visit family back in my country of origin, hearing and reading bad news about pandemic, feeling lonely, working at the University campus which was deserted, no extra activities after work* (Female, 39)

One distinguishable difference to other classes was the high prevalence of fear related to COVID-19. This fear prevented many people to exercise and impacted on their mental health.

*Lack of motivation to go out, scared in case of infection (*Female, 56)

##### 3.1.1.6. Class 5: remain inactive, distressed and lonely (*n* = 18)

Almost half (47%, *n* = 18) of participants in the Inactive, Distressed and Lonely class remained there after the lockdown ended. These individuals reported highest prevalence of mental health issues and alcohol consumption in comparison with the other classes. Everyone felt lonely and spent more than 2 h/day using screens with the majority being physically inactive.

Response to the open-ended questions suggests that people in this class lost their motivation in general during the lockdown and experienced lethargy in every aspect of their lives (e.g., exercise, food, and social interaction). Many mentioned that COVID-19 had a huge impact on their mental health. For example, people were feeling more fatigued in general and were struggling to get back to their normal routines.

*Still haven't gone back to training 100% due to general uncertainty/anxiety/disruption in training/training offered is less then pre COVID leading to a “why bother” attitude* (Female, 36)

*Before COVID I was more interested in cooking different meals however now I'm too lazy to cook and will usually go a day or two without eating* (Male, 19)

When asked about positive or negative impacts of the COVID-19 lockdown on mental wellbeing, many mentioned job insecurities, feeling isolated, lonely, or claustrophobic. Many also mentioned the fear of not knowing what would happen. Some, however, also recalled some positive things, such as not feeling pressured to attend social gatherings.

*Lockdown positively affected my mental wellbeing sometimes because I felt like I wasn't under pressure to do things. I didn't have appointments or commitments and that took a lot of anxiety away. What negatively affected my mental health was the existential doom of not knowing what was happening and so many people dying* (Female, 22)

#### 3.1.2. LTA class comparisons

**Figure 3:**
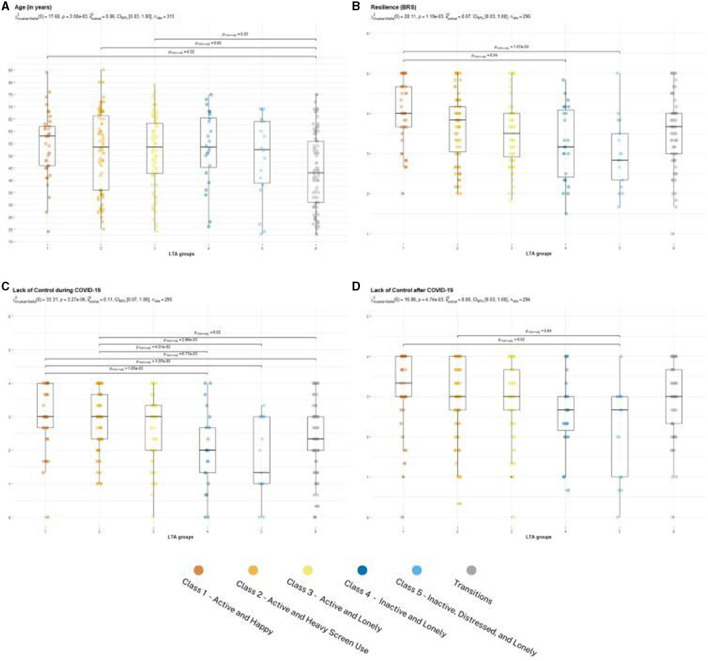
**(A–D)** LTA group comparison Kruskal–Wallis (*p* < 0.05).

The group differences between those who remain in their class or transit between classes were examined by a Kruskal–Wallis test. The results revealed significant differences in age, resilience, and lack of control during and after lockdown, between the LTA classes (Figure 3) (Supplementary Tables S8, S9; Supplementary Figures S1, S2).

The median age was significantly lower in *Transition* group in comparison with groups that remain active (Figure 3A). The highest resilience was detected among individuals who remain *Active and Happy*; the group median was statistically higher in comparison with people who remain inactive (Figure 3B). Similarly, individuals whom either remained *Active and Happy*, or *Active and High Screen Use* felt more in control during the lockdown than individuals who either remained inactive or transitioned between the classes (Figure 3C). When investigating the lack of control after lockdown, only the comparison between *Inactive, Distressed and Lonely* and active classes remained significant (Figure 3D).

In general, people who remained *Active and Happy* or *Active and Heavy Screen Use* reported to be more understandable toward the governmental restrictions and were more aware of their own actions that the other classes. They also tried to maintain control over their personal lives by focusing on keeping up with healthy routines, like exercising and eating.

*I think I compensated by taking as much control over what I could during the lockdown. Health, eating, my living space, how I worked, etc*. (Male, 28, Remain Active and Heavy Screen Use)

On the other hand, people who remained inactive, especially *Inactive, Distressed and Lonely*, seemed to struggle to adapt to lockdown rules, and some reported experiencing fear and lack of control because of the restrictions.

*Restrictions meant had no control. Not being allowed to leave home, the state or the country*

*Quite frightening* (Female, 53, Remain Inactive, Distressed and Lonely)

**Figure 4:**
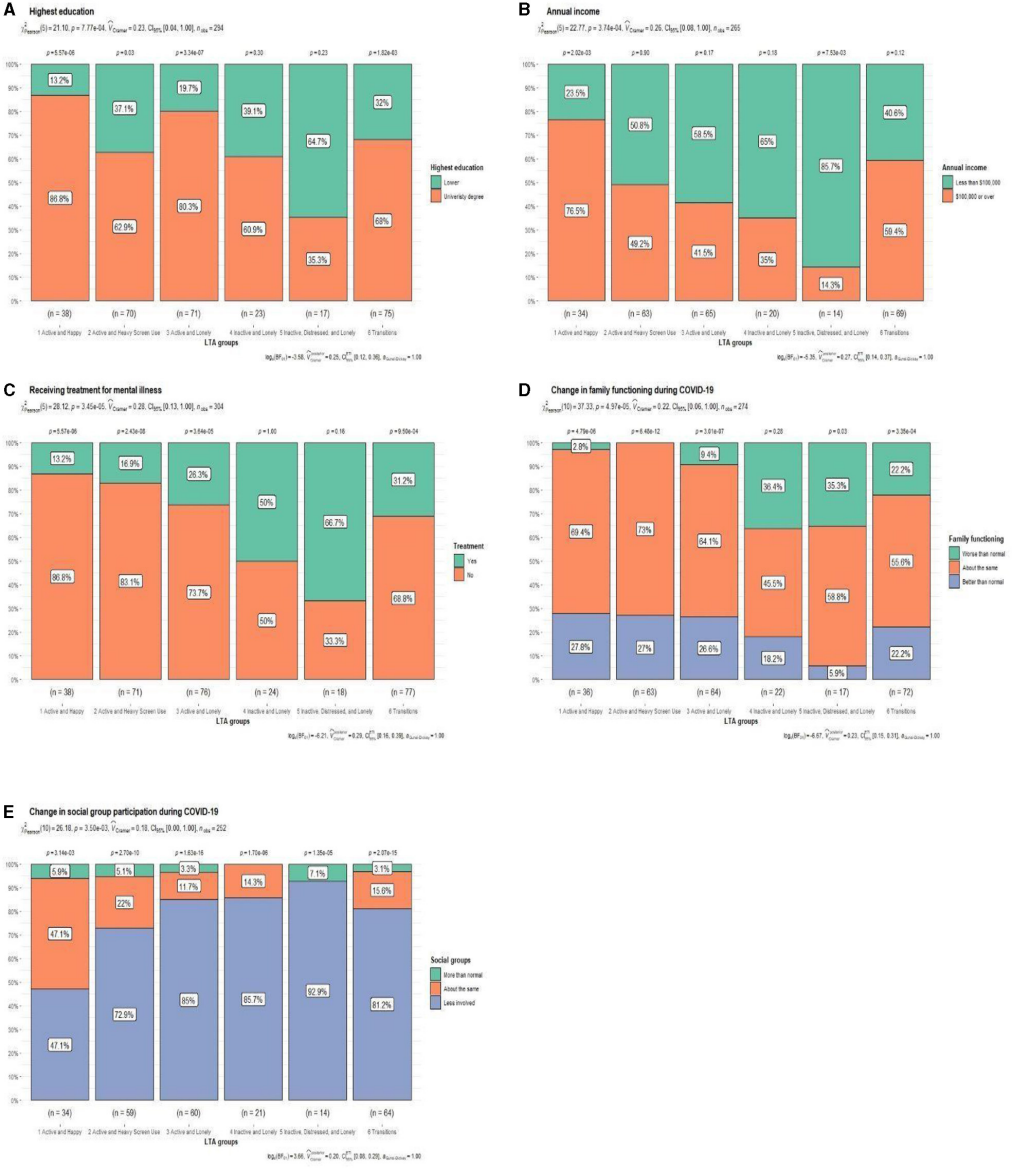
**(A–E)** LTA group comparison chi-square test (*p* < 0.05).

Chi-square tests revealed statistically significant differences in education and household income, existing mental health illnesses, change in social group involvement, and family functioning during COVID-19 (Figure 4). There were no statistically significant differences detected for sex, country of origin or indigenous heritage, or starting period of mental health treatment (Supplementary Table S10; Supplementary Figures S3–S6). Furthermore, the quantitative representation of the multiple responses of open-ended responses regarding effects of COVID-19 and health promotion campaigns were investigated and are discussed in this section. The quantified results are presented in Supplementary Tables S11, S12.

Lower education and annual income were associated with being *Inactive, Distressed and Lonely*. In contrast, people who remained *Active and Happy* were more likely to have a university degree and greater annual income (Figures 4A, B). Consequently, people who remained inactive or felt lonely reported greater changes in their social relations during COVID-19 (Figures 4D, E). People who remained inactive, over one-third, reported worsening family functioning. Indeed, *Inactive, Distressed and Lonely* were more likely than any other group to report negative consequence of the COVID-19 lockdown related to family functioning, e.g., “*forcing to spend time together”* (Supplementary Table S11). On the other hand, staying touch with social groups might be more associated with feeling of loneliness rather than physical activity (Figure 4E). For example, almost half of people who remain *Active and Happy* reported no change in their social relations. In addition, they were most likely to report more positive (e.g., “*more family time”*) than negative effects (e.g., “*difficulties to catch up with family and friends”*) than other classes (Supplementary Table S11).

The classes differed based on the existing mental health issues (Figure 4C). People who were inactive classes, more than half, reported receiving treatment for mental health issues, highest being among people who remained *Inactive, Distressed and Lonely*. When asking participants to comment or describe any positive or negative impacts that COVID-19 had on their physical and mental wellbeing (Supplementary Table S11), people who remained inactive reported to be more frustrated toward the governmental restrictions (e.g., “*loss of basic freedom”*). These classes were more likely to raise mental health issues, concerns, and fears as an impact of COVID-19. For example, people who remained *Inactive and Lonely* reported the highest prevalence of increased anxiety and fear. These people seemed to be more worried about COVID-19 itself (i.e., getting sick), change in restrictions, or changes in their personal life (e.g., job insecurity)

*Felt safe so long as no one entered the house and this is when I felt I had little control* (Female, 73, Remain Inactive and Lonely)

*Uncertainty about future, job insecurity* (Female, 46, Remain Inactive and Lonely)

People who remained *Inactive and Lonely* appeared to recall most health promotion campaigns, especially ones related to hygiene (e.g., “washing hands” and “coughing into elbow”), whereas *Inactive, Distressed and Lonely* or *Transition* groups were the most likely to not recall any health promotion campaigns, nor to make any changes to their behavior as a result of these campaigns (Supplementary Table S12).

In general, washing hands and social distancing were the most recalled and effective health campaigns campaign across the classes, with health campaigns encompassing physical activity/alcohol consumption, or domestic violence having less attention. People who remained *Active and Lonely* seemed to have the best response to the health campaigns by reporting the greatest change in their behavior (e.g., “washing hands” and “sanitizer use”) (Supplementary Table S12).

When asking about which health promotion messages should have been provided to the community during COVID-19 lockdown, the most common responses across the groups were “Better access to timely WA specific information via web,” especially along with people who were classified as lonely (Supplementary Table S12). Other most supported campaigns were “*clearer instructions of mask wearing”* or “*physical, nutritional, and mental well-being campaigns*.”

## 4. Discussion

To the best of our knowledge, this is the first study that uses LTA to investigate the behavioral changes in the general population during and after COVID-19 lockdown, whereas previous LTA studies have investigated the change either before and during (3, 4), or before and after (47) COVID-19 lockdown. Using validated and widely utilized psycho-social measures, LTA analysis identified five latent classes with unique combinations of physical activity, leisure screen time use, alcohol consumption, loneliness, and mental distress during COVID lockdown. Two classes were characterized by inactivity, and three classes were characterized as active. The transition between LTA classes post-COVID-19 lockdown provided important insights for protective and risk factors for maintaining health and wellbeing during pandemic conditions, extending the research by Bhoyroo et al. (8) and Piggott et al. (24).

Since the LTA can identify subpopulations based on the behavioral profiles, it can be useful for informing diverse targeted strategies to effectively plan future health promotion campaigns and procedures in future. For example, some of the topics that were identified in the studies by Bhoyroo et al. (8) and Piggott et al. (24) also emerged in the present study as being associated with specific subgroups. For instance, Piggott et al. (24) reported that some people were “*challenged to stay motivated and to find new ways to exercise in general or lacked motivation overall,”* which is most likely be associated with inactive groups from the present study. Similarly, the present study revealed detailed information of the different characteristics of individuals that reported “*no change*” in Bhoyroo et al. (8) study—that is, individuals who “*remain”* in their group in present study. Group-level analysis, such as LTA, reveals different type of trajectories of wellbeing and health behavior that conventional sample- or population-level studies fail to detect. By expanding the methodological approach in COVID-19 studies, we gained valuable information of how different groups within the population are experiencing the health and social impacts of COVID-19. This evidence can inform the reduction in health inequality within society by creating more efficient health responses to future health crises.

Confirming our aim of the study, parts of the community reacted differently in how they managed with the lockdown restrictions but also in their recovery. Being active and not feeling lonely helped people be resilient to the stressful event of COVID-19 lockdown. *Active and Happy* and *Inactive, Distressed and Lonely* classes were distinctly different, emphasizing the importance of physical activity and social support for recovering from COVID-19 lockdown. For example, people who were *Active and Happy* reported higher resilience, sense of control, and reported less existing mental health issues, and fewer changes in their social relations during the lockdown than people who were inactive or lonely. This finding is supported by a large longitudinal study conducted in UK that found loneliness and decreased physical activity were risk factors for worsening mental health during the pandemic among people over 50 (48). Similarly, lower resilience, along with COVID-19-related worries, were reported to moderate the relationship between COVID-19-related loneliness and sleep problems among older population in Iran (49). These findings are highlighting the different needs and behaviors within the population, and therefore the need of more diversified ways to communicate with different subgroups of the public. In fact, Hyland-Wood et al. (50) highlighted the importance of tailored public health messaging for diverse audience in their article encompassing key characteristics and recommendations for effective governmental communication during crisis management, such as COVID-19. As an example, Hyland-Wood et al. (50) listed, e.g., people with disabilities, language barriers, or cultural differences, or age-sensitive public health messaging. Failing to identify some of these subgroups may lead to increased health risks within the community, like spreading the COVID-19 (50).

Our results also examined the impacts of health campaigns during COVID-19. First, the community education programs about the importance of hand hygiene and social distancing were the most recalled and effective health campaigns across all the classes which suggests that this multi-pronged strategy was well-received and successful. However, our study also revealed an overall increase in alcohol intake, loneliness, and mental distress along with decrease in physical activity, yet participants failed to recall these health campaigns. In general, most individuals reported being worried or scared of the unknown circumstances related to COVID-19 or being negatively affected by how the media was reporting COVID-19-related news suggesting there is the need for a more optimistic outlook to the public when advocating the public health messages. This is supported by many people across the groups requesting better access to the local state COVID-19 information and other studies that have discovered that trust in government or other institutes during COVID-19 are associated with improved mental health, sense of control, or lower COVID-related stressors (51–59). For example, Roccatto et al. (56) discovered that participants' perceived control over their lives mediated the association between political trust, mental health, and collective angst and anger. Similarly, Hyland-Wood et al. (50) emphasized the importance of developing and maintaining the public trust by providing clear and transparent public health messaging via trusted platforms (e.g., governmental website) to achieve a long-term success in crisis communication. In future, governments should pay attention to their public health messaging, so it is comprehensive and up to date. It is highly encouraged that the content should also convey positive and assuring, yet firm, information to enhance public's mental health and sense of control during uncertain times. The public health message should focus on emphasizing the sense of togetherness, along with practical examples of how to cope during COVID-19 lockdown (58, 60).

Second, the need of public health messaging was more essential among people who were either inactive or were in the *Transition* class. For example, people who were *Inactive, Distressed and Lonely* seemed to struggle the most but rarely managed to recall any health promotion campaigns nor made changes in their behavior, indicating they are a very challenging group to engage with. On the other hand, people who remained *Inactive and Lonely* were able to recall most health promotion campaigns, yet they were the most fearful class. Both classes that remained inactive were also the most frustrated toward the governmental restrictions, and more prone to raise mental health concerns, feelings of lack of control, and COVID-19-related fears, signifying the need of specific and targeted health promotion messaging. These class behaviors confirm the need for public messaging to be clear and coherent across the governmental and health policies since conflicting communication between these providers has been shown to lead to greater confusion, uncertainty, and fear among the public (61). Additionally, weaker trust in government's response to pandemic has been linked to greater anxiety and lack of control (62), which also enhances the likelihood of conspiracy theories (58, 62, 63).

Many in the inactive and *Transition* classes had the lowest level of feeling in control during the lockdown in comparison with active classes during the lockdown. This difference also remained in *Inactive, Distressed and Lonely* class after lockdown finished, raising concerns since the contemporary health campaigns did not seem to reach nor work within this class. Similarly, as many people who transitioned between the classes after lockdown were generally young, the public health campaigns should consider social-demographic differences when designing the content and distribution of the public health messaging. By investigating and understanding the differences between these class characteristics, we can inform public health messaging to be more aligned with how our target audience consumes such information, and from sources they are more likely to engage with. Future studies will be required to help tease out how best to provide these different sections of the community with the knowledge, skills, and support they need to adapt during crisis and return to a balanced level of mental wellbeing post-crisis. Given the strong association between physical activity and psychological wellbeing (64, 65) and the way this relationship has been observed during COVID lockdown (24), the government and health agencies should provide clear and specific messages to the public about the benefits of physical activity and the types of exercises they can do at home or outside when access to the normal gym and other sport activities are restricted.

### 4.1. Implementations

This study identified two key themes that have important implications for future pandemic public health policy and health promotion campaigns: first, the need for better population-level messaging, to address the broader fear/uncertainly related to COVID-19, keeping physically active, alcohol consumption recommendations, and support for domestic violence and second, new, more targeted health promotions to the sections of the community that are of high risk of adverse physical and mental health outcomes following lifting of the imposed restrictions.

### 4.2. Strengths and limitations

Interpretations of the findings of this study need to consider some limitations inherent in its design. As the study was conceptualized during the COVID lockdown, it did not capture pre-COVID-19 data, and while the earlier study indicated that the post-lockdown data reflect pre-COVID levels, this cannot be certain (8). The study relied on participants to recall their responses to the questionnaire at the time of the survey but also for several months earlier, which could result in a level of recall bias (66). Similarly, while initial power estimates indicated the study acquired a satisfactory sample size, the data may suffer from self-selection bias (67), as three-quarters of the participants were female. Furthermore, the sub-setting of the original survey dataset (8) to those that had completed the majority of the survey's open-ended questions did result in the exclusion of many younger people, which could impact on some of the findings.

Despite these potential shortcomings, the study captured responses from over 310 participants and extended the findings from robust and validated instruments using several open-ended questions. Furthermore, the LTA was able to identify population-based behavioral profiles that may be more useful for informing diverse targeted strategies to effectively plan future health promotion campaigns. Therefore, the present methodological approach provides greater insights than many other pre- and post-COVID studies and offers new information of the impacts of public health messaging in specific subgroups during a lockdown period in Western Australia, where the impact of strict social isolation was not confounded by high rates of disease and mortality.

## 5. Conclusion

The COVID-19 lockdown, which dramatically altered the community's normal social function, resulted in significant changes in people's behavior and mental wellbeing, both during and after the lockdown period. While many in the community demonstrated a resilience to the restrictions and maintained a physically active and healthy lifestyle, many reported more negative behaviors. LTA identified five different classes within the community based on their self-reported physical activity levels, leisure screen time, loneliness, and psychological distress. While some people transitioned from poorer states of health and wellbeing following the lifting of restrictions, others remained inactive, lonely, and, in some cases, distressed. In future, governments need to focus attention to their media content and public health messaging by offering comprehensive and up-to-date information and make sure that the information is accessible across the population. Targeted health campaigns tailored to people who are most at risks are recommended. More positive and supportive public health policies are encouraged to help the community maintain their everyday routine and promote their mental health during possible future lockdowns.

## Data availability statement

The raw data supporting the conclusions of this article will be made available by the authors, without undue reservation.

## Ethics statement

The studies involving human participants were reviewed and approved by HREC at the University of Notre Dame Australia (Ref: 2020-133F). The patients/participants provided their written informed consent to participate in this study.

## Author contributions

JC led the study and oversaw the funding arrangements, ethics application, and questionnaire design and data collection with PC. RB helped with ethics application, questionnaire design, data collection, undertook the original data cleaning, and scoring of instruments. KS undertook the analysis and wrote the first manuscript draft. All authors participated in the manuscript review process and approved the final manuscript.
