# Supplementary material for: Bouncing back from COVID-19: a Western Australian community perspective
Source: Front Public Health. 2023 Aug 4;11:1216027. doi: 10.3389/fpubh.2023.1216027 (PMC10436488; doi:10.3389/fpubh.2023.1216027)
Supplement: Supplementary file 1 [file Data_Sheet_1.pdf]

Supplementary Material

Bouncing back from COVID-19: A Western Australian community perspective

Kiira Karoliina Sarasjärvi*, Paola Chivers, Ranila Bhoyroo & Jim Codde

*** Correspondence:**
Kiira Sarasjärvi
kiira.sarasjarvi@helsinki.fi

Table S1 Data exclusion and comparison based on demographics and main variables.

Order	Variable	Original dataset (n=547)	Excluded (n=234)	Subsample (n=313)	Original vs. Subsample	Excluded vs. Subsample
6.	Gender % (n)					
	Male	25.5 (138)	27.6 (64)	23.9 (74)	$\chi^2(1)$ = 0.26,	$\chi^2(1)$ = .923,
	Female	74.5 (403)	72.4 (168)	76.1 (235)	p=.61	p=.337
	Missing	(6)	(2)	(4)		
5.	Age					
	M (SD)	47.96 (16.78)	45.08 (17.71)	50.11 (15.74)	t(858)=1.85, p=.065	t(467.63)=-3.45,
	Mdn (IQR)	50.00 (27.00)	47.00 (30.00)	52.00 (25.00)	U=79251.5, p=.070	p<.001
	Missing	(0)	(0)	(0)		U=30267.00, p<.001
174.	Education % (n)					
	University degree	69.7 (205)	0	69.7 (205)	-	-
	Lower degree	30.3 (89)	0	30.3 (89)		
	Missing	(253)	(234)	(19)		
187.	Household income % (n)					
	$100,000 or over	50.6 (134)	0	50.6 (134)	-	-
	Less than 100,000	49.4 (131)	0	49.4 (131)		
	Missing/Prefer not to Answer	(234)	(234)	(23)/ (25)		
7.	Born in Australia % (n)					
	No	34.7 (188)	28.9 (67)	39.0 (121)	$\chi^2(1)$ = 1.611, p=.205	$\chi^2(1)$ = 6.038, p=.014
	Yes	65.3 (354)	71.1 (165)	61.0 (189)		
	Missing	(5)	(2)	(3)		
8.	Indigenous % (n)					
	No	(528)	(223)	98.4 (305)	$\chi^2(1)$ = 1.938, p=.164	$\chi^2(1)$ = 1.932, p=.165
	Yes	(13)	(8)	1.6 (13)		
	Missing	(6)	(3)	(3)		
20-21.	Physical activity (after) % (n)					
	Inactive	21.6 (100)	24.7 (38)	20.1 (62)	$\chi^2(1)$ = .263,	$\chi^2(1)$ = 1.29,
	Active	78.4 (363)	75.3 (116)	79.9 (247)	p=.608	p=.256
	Missing	(84)	(80)	(4)		
	Physical activity (during)					
	Inactive	35.2 (161)	36.2 (54)	34.7 (107)	$\chi^2(1)$ = .019,	$\chi^2(1)$ = .099,
	Active	64.8 (296)	63.8 (95)	65.3 (201)	p=.889	p=.753
	Missing	(90)	(85)	(5)		
29-30.	Screen time (after) % (n)					
	Less than 2h/day	65.5 (266)	63.4 (64)	66.2 (202)	$\chi^2(1)$ = .039,	$\chi^2(1)$ = .275,
	More than 2h/day	34.5 (140)	36.6 (37)	33.8 (103)	p=.843	p=.600
	Missing	(141)	(133)	(8)		
	Screen time (during)					

	Less than 2h/day	53.2 (216)	52.5 (53)	53.4 (163)	$\chi^2(1) = .004$,	$\chi^2(1) = .029$,
	More than 2h/day	46.8 (190)	47.5 (48)	46.6 (142)	p=.949	p=.866
	Missing	(141)	(133)	(8)		
47-48.	Fast food consumption (after) % (n)					
	Not at all	56.0 (211)	41.1 (30)	59.5 (181)	$\chi^2(1) = .879$,	$\chi^2(1) = 8.125$,
	At least once a week	44.0 (166)	58.9 (43)	40.5 (123)	p=.349	p=.004
	Missing	(170)	(161)	(9)		
	Fast food consumption (during)					
	Not at all	58.4 (220)	49.3 (36)	60.5 (184)	$\chi^2(1) = .329$,	$\chi^2(1) = 3.045$,
	At least once a week	41.6 (157)	50.7 (37)	39.5 (120)	p=.566	p=.081
	Missing	(170)	(161)	(9)		
51-52.	Alcohol intake day (after) % (n)					
	Less than 4 std.drinks	94.2 (343)	88.6 (62)	95.6 (281)	$\chi^2(1) = .615$,	$\chi^2(1) = 5.106$,
	More than 4 std.drinks	5.8 (21)	11.4 (8)	4.4 (13)	p=.433	p=.024
	Missing	(183)	(164)	(19)		
	Alcohol intake day (during)					
	Less than 4 std.drinks	91.8 (335)	92.9 (65)	91.5 (270)	$\chi^2(1) = .014$,	$\chi^2(1) = 0.133$,
	More than 4 std.drinks	8.2 (30)	7.1 (5)	8.5 (25)	p=.906	p=.715
	Missing	(182)	(164)	(18)		
55-82.	Psychological distress (after) K-6 % (n)					
	No probable	90.4 (320)	83.7 (41)	91.5 (279)	$\chi^2(1) = .231$,	$\chi^2(1) = 2.960$,
	Probable disorder	9.6 (34)	16.3 (8)	8.5 (26)	p=.631	p=.085
	Missing	(193)	(185)	(8)		
	Psychological distress (during) K-6					
	No probable	87.3 (302)	84.4 (38)	87.7 (264)	$\chi^2(1) = .027$,	$\chi^2(1) = .376$,
	Probable disorder	12.7 (44)	15.6 (7)	12.3 (37)	p=.871	p=.540
	Missing	(201)	(189)	(12)		
144-149.	Loneliness (after) % (n)					
	No	45.2 (135)	0	45.2 (135)	-	-
	Yes	54.8 (164)	0	54.8 (164)		
	Missing	(248)	(234)	(14)		
	Loneliness (during)					
	No	30.8 (91)	0	30.8 (91)	-	-
	Yes	69.2 (204)	0	69.2 (204)		
	Missing	(252)	(234)	(18)		
130-133	Treating any mental health issues					
	No	72.0 (219)	0	72.0 (219)	-	-
	Yes	28.0 (85)	0	28.0 (85)		
	Missing	(234)	(234)	(9)		
134.	Starting period of treatment % (n)					
	Before COVID-19 lockdown	75.0 (60)	0	75.0 (60)	-	-
	During COVID-19 lockdown	16.3 (13)	0	16.3 (13)		
	After COVID-19 lockdown	8.8 (7)	0	8.8 (7)		
	Total	(80)	(0)	(80)		

159-164.	Brief Resilience Scale (BRS)					
	Mean (SD)	3.57 (0.87)	-	3.57 (0.87)	-	-
	N	296	0	296		
136-141	Lack of Control (3-items) (during)					
	Mean (SD)	2.92 (0.94)	-	2.92 (0.94)	-	-
	N	294	0	294		
	Lack of Control (3-item) (after)					
	Mean (SD)	2.58 (1.03)	-	2.58 (1.03)		
	N	295	0	295		
153-156	Family functioning in general (before COVID-19)					
	Mean (SD)	2.33 (0.66)	-	2.33 (0.66)	-	-
	N	271	0	271		
157.	Change in family functioning during COVID-19 % (n)					
	Better than normal	23.7 (65)	0	23.7 (65)	-	-
	About the same	62.8 (172)	0	62.8 (172)		
	Worse than normal	13.5 (37)	0	13.5 (37)		
	Missing	(273)	(234)	(39)		
150.	Number of social groups before COVID-19 % (n)					
	None	(46)	0	15.4 (46)	-	-
	One or two groups	(97)	0	32.4 (97)		
	Three or more	(156)	0	52.2 (156)		
	Missing	(248)	(234)	(14)		
	M (SD)	2.97 (2.83)	-	2.97 (2.83)		
	N	299	0	299		
	Change in social group involvement during COVID-19 % (n)					
	Less involved	76.6 (193)	0	76.6 (193)	-	-
	About the same	19.4 (49)	0	19.4 (49)		
	More involved	4.0 (10)	0	4.0 (10)		
	Missing	(295)	(234)	(61)		

M = mean, (SD=standard deviation)
Mdn = median, (IQR=interquartile range)

Table S2 Model with 5 Latent Class – used in the present study.

**Indicators:**	Class 2 - Active and heavy screen use	Class 1 - Active and happy	Class 5 - Inactive, distressed and lonely	Class 3- Active and lonely	Class 4 - Inactive and lonely
Physical activity					
Inactive	0.1241	0.2497	**0.7191**	0.00	**0.7663**
Active	**0.8759**	**0.7503**	0.2809	**1.00**	0.2337
Leisure screentime					
Less than 2h/day	0.00	**0.9650**	0.00	**0.9872**	**0.9788**
More than 2h/day	**1.00**	0.0350	**1.00**	0.0128	0.0212
Alcohol intake					
Less than 4 drinks/day	**0.9693**	**1.00**	**0.8036**	**0.9703**	**0.8398**
More than 4 drinks/day	0.0307	0.00	0.1964	0.0297	0.1602
Psychological distress (K-6)					
No	**0.9743**	**1.00**	**0.6164**	**0.9033**	**0.8386**
Yes	0.0257	0.00	0.3836	0.0967	0.1614
Loneliness					
No	0.4650	**1.00**	0.00	0.2712	0.0383
Yes	**0.5350**	0.00	**1.00**	**0.7288**	**0.9617**
**Prevalence of status at**					
During lockdown	0.2967	0.1157	0.1555	0.2558	0.1764
After lockdown	0.2460	0.2173	0.0817	0.3357	0.1193
**Transition from during (rows) to after lockdown (columns)**	Active and heavy screen use	Active and happy	Inactive, distressed and lonely	Active and lonely	Inactive and lonely
C2 – Active and heavy screen use	**0.771**	0.150	0.011	0.068	-
C1 – Active and happy	-	**1.000**	-	-	-
C5 – Inactive, distressed and lonely	0.112	0.096	**0.470**	0.239	0.084
C3 – Active and lonely	-	0.099	-	**0.901**	-
C4 – Inactive and lonely	-	0.096	0.031	0.270	**0.603**

Bolded: Highest Rho (item-response probability) and Tau (transition probabilities) estimates
Model with 5 latent classes: Log-likelihood = -1301.66, G-squared = 328.12, AIC = 426.12, **BIC = 609.68**, df =974

Table S3 Model with 2 Latent Class

**Indicators:**	Group1	Group2
Physical activity		
Inactive	0.1307	0.5366
Active	0.8693	0.4634
Leisure screentime		
Less than 2h/day	0.6676	0.4696
More than 2h/day	0.3324	0.5304
Alcohol intake		
Less than 4 drinks/day	0.9827	0.8501
More than 4 drinks/day	0.0173	0.1499
Psychological distress (K-6)		
No	0.9790	0.7410
Yes	0.0210	0.2590
Loneliness		
No	0.5670	0.0345
Yes	0.4330	0.9655
**Prevalence of status at**		
During lockdown	0.5783	0.4217
After lockdown	0.7170	0.2830
**Transition from during *(rows)* to after lockdown *(columns)***	Group1	Group2
Group1	1.00	-
Group2	0.3289	0.6711

Bolded: Highest Rho (item-response probability) and Tau (transition probabilities) estimates

Model with 2 latent classes: Log-likelihood = -1426.57, G-squared = 577.96, AIC = 603.96, BIC = 652.66, df = 1 010

Table S4 Model with 3 Latent Class

**Indicators:**	Group1	Group2	Group3
Physical activity			
Inactive	-	0.1788	0.7929
Active	1.000	0.8212	0.2971
Leisure screentime			
Less than 2h/day	0.6430	0.6456	0.4947
More than 2h/day	0.3570	0.3544	0.5053
Alcohol intake			
Less than 4 drinks/day	1.00	0.9734	0.8198
More than 4 drinks/day	-	0.0266	0.1802
Psychological distress (K-6)			
No	0.9334	0.9774	0.7654
Yes	0.0666	0.0226	0.2346
Loneliness			
No	0.1453	1.00	0.0270
Yes	0.8547	-	0.9730
**Prevalence of status at**			
During lockdown	0.3679	0.2477	0.3844
After lockdown	0.3834	0.3823	0.2343
**Transition from during *(rows)* to after lockdown *(columns)***	Group1	Group2	Group3
Group1	0.8091	0.1909	-
Group2	-	0.9545	0.0455
Group3	0.2232	0.1966	0.5803

Bolded: Highest Rho (item-response probability) and Tau (transition probabilities) estimates
Model with 3 latent classes: Log-likelihood = -1389.30, G-squared = 503.42, AIC = 549.42, BIC = 635.58, df = 1 000

Table S5 Model with 4 Latent Class

**Indicators:**	Group1	Group2	Group3	Group4
Physical activity				
Inactive	0.0362	1.000	0.6609	0.0495
Active	0.9638	-	0.3391	0.9505
Leisure screentime				
Less than 2h/day	0.6322	0.7457	0.4339	0.6485
More than 2h/day	0.3678	0.2543	0.5661	0.3515
Alcohol intake				
Less than 4 drinks/day	1.000	1.000	0.7677	0.9784
More than 4 drinks/day	-	-	0.2323	0.0216
Psychological distress (K-6)				
No	0.9480	1.000	0.6877	0.9701
Yes	0.0520	-	0.3123	0.0299
Loneliness				
No	0.0004	0.5034	0.0367	1.000
Yes	0.9996	0.4966	0.9633	-
**Prevalence of status at**				
During lockdown	0.3500	0.1080	0.3003	0.2417
After lockdown	0.3365	0.0560	0.1935	0.4140
**Transition from during *(rows)* to after lockdown *(columns)***	Group1	Group2	Group3	Group4
Group1	0.6661	0.0033	-	0.3306
Group2	-	0.5075	-	0.4925
Group3	0.2422	-	0.6201	0.1377
Group4	0.1270	-	0.0300	0.8430

Bolded: Highest Rho (item-response probability) and Tau (transition probabilities) estimates

Model with 4 latent classes: Log-likelihood = -1377.59, G-squared = 479.99, AIC = 549.99, BIC = 681.10, df = 988

Table S6 Model with 6 Latent Class

**Indicators:**	Group1	Group2	Group3	Group4	Group5	Group6
Physical activity						
Inactive	0.1802	1.000	0.6892	0.0675	0.2738	0.0746
Active	0.8198	-	0.3108	0.9325	0.7262	0.9274
Leisure screentime						
Less than 2h/day	-	0.7306	0.1284	0.0358	1.000	1.000
More than 2h/day	1.000	0.2694	0.8716	0.9641	-	-
Alcohol intake						
Less than 4 drinks/day	0.9967	1.000	0.7021	0.9721	0.9355	0.9670
More than 4 drinks/day	0.0033	-	0.2979	0.0279	0.0645	0.0330
Psychological distress (K-6)						
No	1.000	1.000	0.4947	0.9742	0.8687	0.9700
Yes	-	-	0.5053	0.0258	0.1313	0.0300
Loneliness						
No	0.000	0.5692	-	1.000	.	0.8216
Yes	1.000	0.4308	1.000	-	1.000	0.1784
**Prevalence of status at**						
During lockdown	0.1887	0.0840	0.1463	0.1288	0.2845	0.1676
After lockdown	0.1362	0.0440	0.0827	0.1275	0.2565	0.3531
**Transition from during (rows) to after lockdown (columns)**	Group1	Group2	Group3	Group4	Group5	Group6
Group1	0.5298	-	-	0.2119	0.0916	0.1566
Group2	-	0.5237	0.0214	-	-	0.4549
Group3	0.1941	-	0.4410	-	0.1734	0.1914
Group4	0.0459	-	0.1271	0.6790	-	0.1480
Group5	-	-	-	-	0.7516	0.2482
Group6	-	-	-	-	-	1.000

Bolded: Highest Rho (item-response probability) and Tau (transition probabilities) estimates
Model with 6 latent classes: Log-likelihood = -1288.40, G-squared = 301.60, AIC = 431.60, BIC = 675.10, df =958

Table S7 Model with 7 Latent Class

**Indicators:**	Group1	Group2	Group3	Group4	Group5	Group6	Group7
Physical activity							
Inactive	0.1940	1.000	0.7369	0.0656	0.2557	0.0648	0.2539
Active	0.8060	-	0.2631	0.9344	0.7443	0.9352	0.7461
Leisure screentime							
Less than 2h/day	-	0.7311	0.1277	0.0358	1.000	1.000	0.7431
More than 2h/day	1.000	0.2689	0.8723	0.9642	-	-	0.2568
Alcohol intake							
Less than 4 drinks/day	0.9967	1.000	0.7137	0.9856	0.9672	1.000	-
More than 4 drinks/day	0.0033	-	0.2863	0.0144	0.0328	-	1.000
Psychological distress (K-6)							
No	1.000	1.000	0.4120	0.9738	0.8641	0.9689	1.000
Yes	-	-	0.5880	0.0262	0.1359	0.0311	-
Loneliness							
No	-	0.5265	-	1.000	-	0.8212	0.3984
Yes	1.000	0.4735	1.000	-	1.000	0.1788	0.6016
**Prevalence of status at**							
During lockdown	0.1970	0.0933	0.1271	0.1249	0.2738	0.1609	0.0230
After lockdown	0.1421	0.0484	0.0714	0.1273	0.2431	0.3410	0.0267
**Transition from during (rows) to after lockdown (columns)**	Group1	Group2	Group3	Group4	Group5	Group6	Group7
Group1	0.5353	-	-	0.2028	0.1016	0.1604	-
Group2	-	0.5193	0.0358	-	-	0.4449	-
Group3	0.2313	-	0.4472	-	0.1290	0.1617	0.0307
Group4	0.0577	-	0.0898	0.6992	-	0.1532	-
Group5	-	-	-	-	0.7549	0.2451	-
Group6	-	-	-	-	-	1.000	-
Group7	-	-	-	-	-	0.0093	0.9907

Bolded: Highest Rho (item-response probability) and Tau (transition probabilities) estimates
Model with 7 latent classes: Log-likelihood = -1269.28, G-squared = 263.36, AIC = 429.36, BIC = 740.30, df =940

Table S8 LTA group comparisons: Kruskal-Wallis test

Groups	Active and heavy Screen use	Active and Happy	Inactive, distressed and lonely	Active and lonely	Inactive and lonely	All transitions	
% (n)	23.8% (63)	12.1% (32)	5.3% (14)	24.9% (66)	7.9% (21)	26.0% (69)	
Variable	Mean (SD)<br>Median (IQR)	Mean (SD)<br>Median (IQR)	Mean (SD)<br>Median (IQR)	Mean (SD)<br>Median (IQR)	Mean (SD)<br>Median (IQR)	Mean (SD)<br>Median (IQR)	Statistical comparison
Age	51.9 (16.3)<br>53.0 (32.0)	55.4 (13.3)<br>58.0 (17.0)	49.1 (17.3)<br>50.5 (27.0)	52.7 (15.0)<br>55.0 (22)	52.1 (15.4)<br>53.0 (25.0)	44.6 (15.7)<br>43.0 (29.0)	H(5) = 17.6,<br>p = .003,<br>p^a = .045
Resilience (BRS)	3.7 (0.8)<br>3.8 (1.2)	4.0 (0.8)<br>4.0 (1.0)	3.1 (0.9)<br>2.9 (1.5)	3.5 (0.9)<br>3.6 (1.2)	3.3 (0.9)<br>3.3 (1.5)	3.5 (0.8)<br>3.7 (1.0)	H(5) = 20.1,<br>p = .001,<br>p^a = .015
Number of social groups before C19 (with outlier)	2.7 (2.2)<br>2.0 (3.0)	3.8 (3.1)<br>3.0 (2.8)	4.1 (7.8)<br>1.5 (4.5)	2.7 (1.7)<br>3.0 (3.0)	2.4 (1.5)<br>3.0 (2.0)	2.9 (2.3)<br>3.0 (2.5)	H(5) = 1.6,<br>p = .895
Number of Social groups before C19 (no outlier)	3.0 (2.6)<br>3.0 (4.0)	3.5 (3.0)<br>3.3 (3.3)	2.4 (2.3)<br>2.0 (3.0)	2.6 (1.7)<br>3.0 (3.0)	2.8 (2.1)<br>3.0 (2.0)	2.8 (2.3)<br>2.0 (3.0)	H(5) = 2.5,<br>p = .780
Family functioning in general	2.4 (0.7)<br>2.5 (1.0)	2.6 (0.5)<br>2.8 (0.8)	2.0 (0.6)<br>2.1 (1.1)	2.2 (0.7)<br>2.3 (1.0)	2.1 (0.6)<br>2.3 (1.1)	2.4 (0.7)<br>2.5 (1.0)	H(5) = 13.0,<br>p = .023,<br>p^a = .345
Lack of control during lockdown	3.0 (0.9)<br>3.0 (1.3)	3.1 (1.0)<br>3.0 (1.3)	1.8 (1.2)<br>1.5 (2.0)	2.6 (0.9)<br>3.0 (1.3)	2.0 (1.1)<br>2.0 (1.5)	2.4 (1.0)<br>2.3 (1.0)	H(5) = 33.3,<br>p < .001,<br>p^a < .015
Lack of control after lockdown	3.1 (0.9)<br>3.3 (1.3)	3.1 (1.0)<br>3.5 (1.3)	2.2 (1.2)<br>2.7 (2.1)	3.0 (0.9)<br>3.0 (1.0)	2.6 (1.0)<br>2.7 (1.2)	3.0 (0.8)<br>3.0 (1.3)	H(5) = 16.9,<br>p = .005,<br>p^a = .075

p^a = adjusted p-value

Table S9 Post-Hoc comparisons based on the LTA groups Dunn's pairwise tests and Bonferroni correction

**Post-Hoc comparison**		**Age**	**Resilience**	**Family Functioning**	**Lack of control during lockdown**	**Lack of control after lockdown**
Active and heavy screen use vs.	Active and happy	D = -10.5<br>p = .563<br>P^a = 1.00	D = -32.7<br>p = .057<br>P^a = .859	D = -19.6<br>P = .240<br>p^a = 1.00	D = -13.6<br>p = .425<br>p^a = 1.00	D = -10.6<br>p = .533<br>p^a = 1.00
	Inactive, distressed and lonely	D = 11.5<br>p = .630<br>P^a = 1.00	*D = 66.2*<br>*p = .004*<br>*P^a =.062*	D = 49.2<br>P = .030<br>p^a = .455	**D = 84.5**<br>**p = .000**<br>**p^a =.003**	**D = 68.1**<br>**p = .003**<br>**p^a = .040**
	Active and lonely	D = 2.5<br>p = .868<br>P^a = 1.00	D = 16.2<br>p = .260<br>P^a = 1.00	D = 17.3<br>P = .197<br>p^a = 1.00	D = 26.1<br>p = .066<br>p^a =.995	D = 9.8<br>p = .488<br>p^a = 1.00
	Inactive and lonely	D = -6.1<br>p = .774<br>P^a = 1.00	D = 34.5<br>p = .092<br>P^a = 1.00	D = 34.8<br>P = .072<br>p^a = 1.00	**D = 72.5**<br>**p = .000**<br>**p^a =.006**	D = 51.2<br>p = .011<br>p^a = .168
	All transitions	**D = 46.2**<br>**p = .001**<br>**P^a =.021**	D = 11.4<br>p = .420<br>P^a = 1.00	D = -.057<br>P = .997<br>p^a = 1.00	**D = 44.1**<br>**p = .002**<br>**p^a = .027**	D = 13.8<br>p = .323<br>p^a = 1.00
Active and happy vs.	Inactive, distressed and lonely	D = 22.0<br>p = .396<br>P^a = 1.00	**D = 98.9**<br>**p = .00**<br>**P^a =.001**	*D = 68.8*<br>*P = .005*<br>*p^a =.081*	**D = 98.1**<br>**p = .000**<br>**p^a =.001**	**D = 78.7**<br>**p = .001**<br>**p^a = .020**
	Active and lonely	D = 13.0<br>p = .470<br>P^a = 1.00	*D = 48.9*<br>*p = .004*<br>*P^a = .065*	D = 36.8<br>P = .026<br>p^a = .390	D = 39.7<br>p = .019<br>p^a =.285	D = 20.4<br>p = .226<br>p^a = 1.00
	Inactive and lonely	D = 4.4<br>p = .852<br>P^a = 1.00	**D = 67.2**<br>**p = .003**<br>**P^a =.043**	D = 54.3<br>P = .012<br>p^a = .181	**D = 86.1**<br>**p = .000**<br>**p^a =.002**	*D = 61.7*<br>*p = .005*<br>*p^a = .080*
	All transitions	**D = 56.7**<br>**p = .001**<br>**P^a =.020**	D = 44.1<br>p = .009<br>P^a =.138	D = 19.5<br>P = .235<br>p^a = 1.00	**D = 57.7**<br>**p = .001**<br>**p^a = .009**	D = 24.3<br>p = .144<br>p^a = 1.00
Inactive, distressed and lonely vs.	Active and lonely	D = -9.0<br>p = .704<br>P^a = 1.00	D = -50.0<br>p = .030<br>P^a = 451	D = -32.0<br>P = .158<br>p^a = 1.00	*D = -58.4*<br>*p = .010*<br>*p^a =.157*	*D = -58.3*<br>*p = .010*<br>*p^a =.151*
	Inactive and lonely	D = -17.6<br>p = .533<br>P^a = 1.00	D = -31.6<br>p = .246<br>P^a = 1.00	D = -14.4<br>P = .588<br>p^a = 1.00	D = -12.0<br>p = .658<br>p^a = 1.00	D = -16.9<br>p = .528<br>p^a = 1.00
	All transitions	D = 34.7<br>p = .139<br>P^a = .100	D = -54.8<br>p = .017<br>P^a =.248	D = -49.3<br>P = .029<br>p^a =.434	D = -40.4<br>p = .076<br>p^a = 1.00	*D = -54.3*<br>*p = .016*<br>*p^a = .236*
Active and lonely vs.	Inactive and lonely	D = -8.6<br>p = .685<br>P^a = 1.00	D = 18.3<br>p = .370<br>P^a = 1.00	D = 17.5<br>P = .362<br>p^a = 1.00	D = 46.4<br>p = .022<br>p^a = .329	D = 41.4<br>p = .040<br>p^a = .596
	All transitions	**D = 43.8**<br>**p = .002**<br>**P^a =.033**	D = -4.8<br>p = .731<br>P^a =1.00	D = -17.3<br>P = .186<br>p^a = 1.00	D = 18.00<br>p = .196<br>p^a = 1.00	D = 3.9<br>p = .775<br>p^a = 1.00
Inactive and lonely	All transitions	D = 53.4<br>p = .012<br>P^a =.185	D = -23.2<br>p = .253<br>P^a = 1.00	D = -34.9<br>P = .069<br>p^a = 1.00	D = -28.4<br>p = .159<br>p^a = 1.00	D = -37.4<br>p = .061<br>p^a = .911

a. adjusted p-value Bonferroni correction, **statistically significant (p<.05) in bold**, *statistical trend p < .10 in italics*

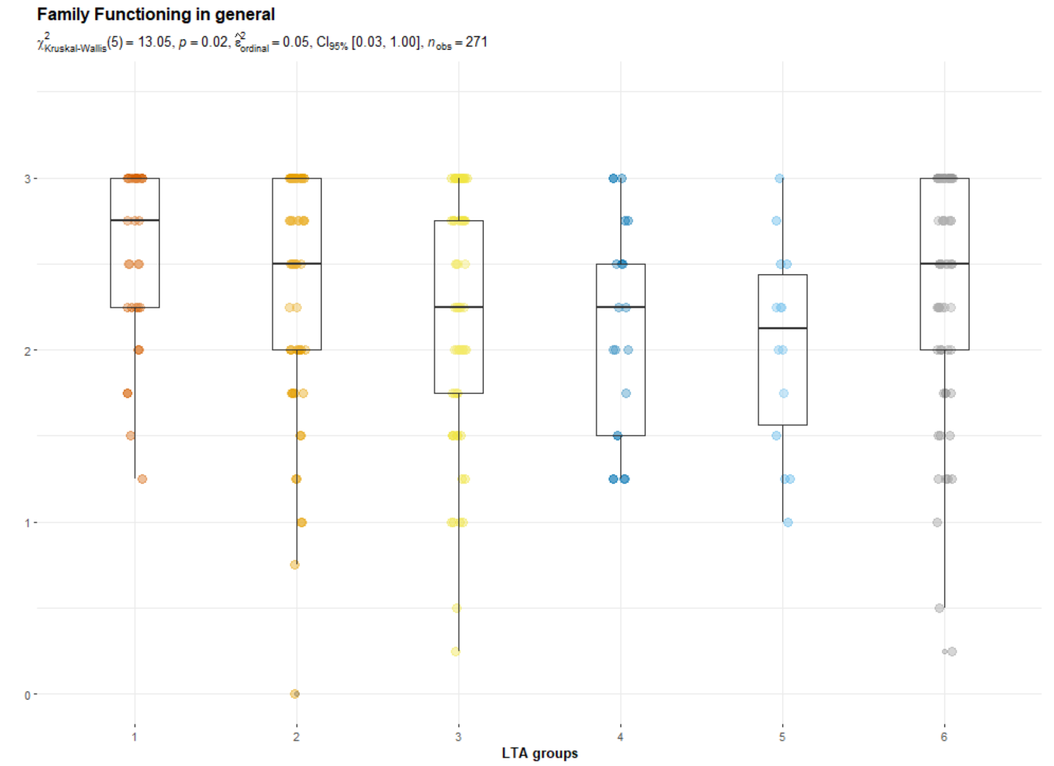

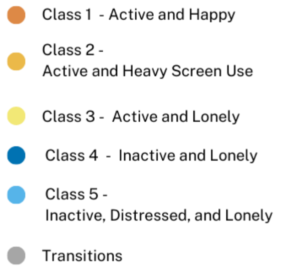

Figure S2 Family functioning in general (ns.)

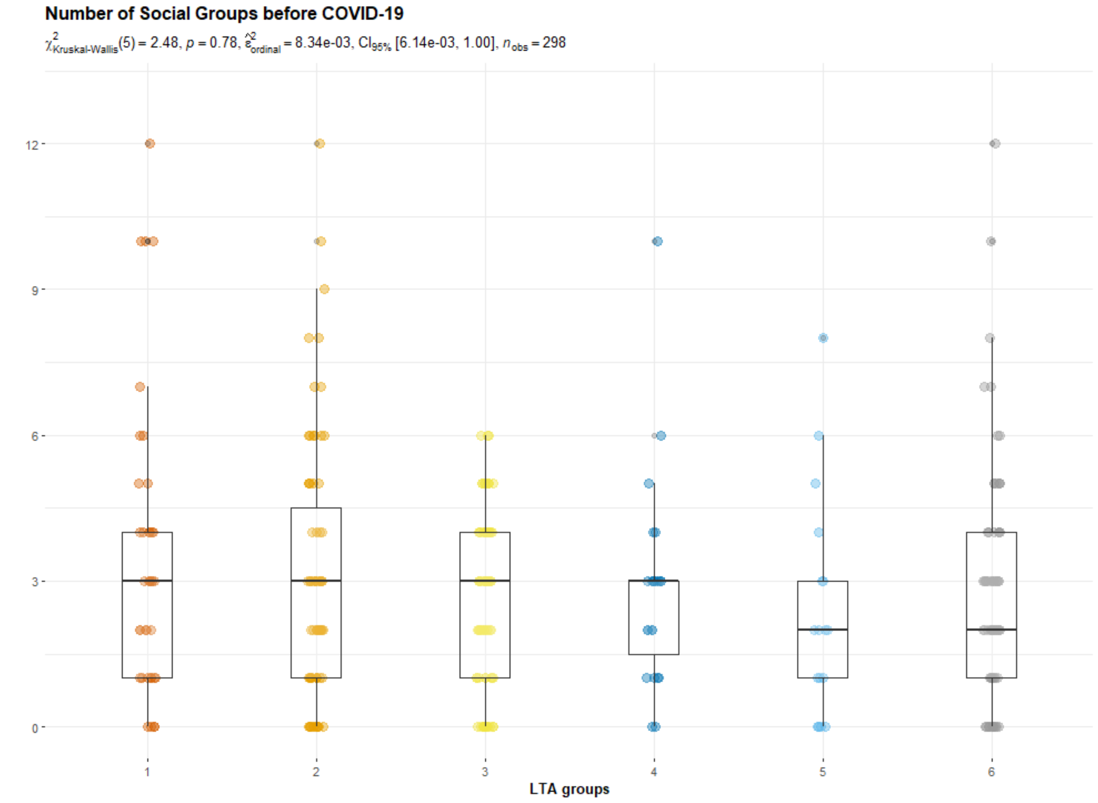

Figure S1 LTA group comparison – Number of social groups before COVID-19 (ns.)

Table S10 LTA group comparisons: Chi-square test.

Variable % row/ column (n)	Active and heavy screen use	Active and happy	Inactive, distressed and lonely	Active and lonely	Inactive and lonely	All transitions	Total	Comparisons
**Sex**								
Male	24.3/ 25.4 (18)	14.9 / 28.9 (11)	5.4 / 22.2 (4)	25.7 / 25.0 (19)	4.1 / 12.5 (3)	25.7 / 23.2 (19)	100 (74)	$\chi^2(5) = 2.428$,
Female	22.6/ 74.6 (53)	11.5 / 71.1 (27)	6.0 / 77.8 (14)	24.3 / 75.0 (57)	8.9 / 87.5 (21)	26.8 / 76.8 (63)	100 (235)	$p = .787$
Total	23.0/ 100 (71)	12.3 / 100 (38)	5.8 / 100 (18)	24.6 / 100 (76)	7.8 / 100 (24)	26.5 / 100 (82)	100 (309)	
**Born in Australia**								
No	22.3 / 37.5 (27)	13.2 / 43.2 (16)	5.0 / 33.3 (6)	24.8 / 39.5 (30)	5.0 / 25.0 (6)	29.8 / 43.4 (36)	100 (121)	$\chi^2(5) = 3.242$,
Yes	22.8 / 62.5 (45)	11.1 / 56.8 (21)	6.3 / 66.7 (12)	24.3 / 60.5 (46)	9.5 / 75.0 (18)	24.9 / 56.6 (47)	100 (189)	$p = .663$
Total	23.3 / 100 (72)	11.9 / 100 (37)	5.8 / 100 (18)	24.5 / 100 (76)	7.7 / 100 (24)	26.8 / 100 (83)	100 (310)	
**Indigenous**								
No	23.0 / 97.2 (70)	12.5 / 100 (38)	5.6 / 94.4 (17)	24.9 / 100 (76)	7.9 / 100 (24)	26.2 / 97.6 (80)	100 (305)	$\chi^2(5) = 4.994$,
Yes	40.0 / 2.8 (2)	0 / 0 (0)	20.0 / 5.6 (1)	0 / 0 (0)	0 / 0 (0)	40.0 / 2.4 (2)	100 (5)	$p = .417$
Total	23.3 / 100 (72)	12.3 / 100 (38)	5.8 / 100 (18)	24.5 / 100 (76)	7.7 / 100 (24)	26.5 / 100 (82)	100 (310)	
**Highest education (University degree)**								
Yes	21.5 / 62.9 (44)	16.1 / 86.8 (33)	2.9 / 35.3 (6)	27.8 / 80.3 (57)	6.8 / 60.9 (14)	24.9 / 68.0 (51)	100 (205)	$\mathbf{\chi^2(5) = 21.095}$**,**
No	29.2 / 37.1 (26)	5.6 / 13.2 (5)	12.4 / 64.7 (11)	15.7 / 19.7 (14)	10.1 / 39.1 (9)	27.0 / 32.0 (24)	100 (89)	$\mathbf{p < .001}$
Total	23.8 / 100 (70)	12.9 / 100 (38)	5.8 / 100 (17)	24.1 / 100 (71)	7.8 / 100 (23)	25.5 / 100 (75)	100 (294)	
**Household income $100,000 per year**								
More	23.1 / 49.2 (31)	19.4 / 76.5 (26)	1.5 / 14.3 (2)	20.1 / 41.5 (27)	5.2 / 35.0 (7)	30.6 / 59.4 (41)	100 (134)	$\mathbf{\chi^2(5) = 22.768}$**,**
Less	24.4 / 50.8 (32)	6.1 / 23.5 (8)	9.2 / 85.7 (12)	29.0 / 58.5 (38)	9.9 / 65.0 (13)	21.4 / 40.6 (28)	100 (131)	$\mathbf{p < .001}$
Total	23.8 / 100 (63)	12.8 / 100 (34)	5.3 / 100 (14)	24.5 / 100 (65)	7.5 / 100 (20)	26.0 / 100 (69)	100 (265)	
**Treatment for mental health issues**								
No	26.9 / 83.1 (59)	15.1 / 86.8 (33)	2.7 / 33.3 (6)	25.6 / 73.7 (56)	5.5 / 50.0 (12)	24.2 / 68.8 (53)	100 (219)	
Yes	14.1 / 16.9 (12)	5.9 / 13.2 (5)	14.1 / 66.7 (12)	23.5 / 26.3 (20)	14.1 / 50.0 (12)	28.2 / 31.2 (24)	100 (85)	$\mathbf{\chi^2(5) = 28.116}$**,**
Total	23.4 / 100 (71)	12.5 / 100 (38)	5.9 / 100 (18)	25.0 / 100 (76)	7.9 / 100 (24)	25.3 / 100 (77)	100 (304)	$\mathbf{p < .001}$
**Starting date (COVID-19)**								
Before	15.0 / 75.0 (9)	6.7 / 100 (4)	15.0 / 81.8 (9)	25.0 / 83.3 (15)	10.0 / 50.0 (6)	28.3 / 73.9 (17)	100 (60)	$\chi^2(10) = 6.459$,
During	15.4 / 16.7 (2)	0 / 0 (0)	7.7 / 9.1 (1)	15.4 / 11.1 (2)	30.8 / 33.3 (4)	30.8 / 17.4 (4)	100 (13)	$p = .775$
After	14.3 / 8.3 (1)	0 / 0 (0)	14.3 / 9.1 (1)	14.3 / 5.6 (1)	28.6 / 16.7 (2)	28.6 / 8.7 (2)	100 (7)	
Total	15.0 / 100 (12)	5.0 / 100 (4)	13.8 / 100 (11)	22.5 / 100 (18)	15.0 / 100 (12)	28.7 / 100 (23)	100 (80)	
**Change in family functioning during COVID-19**								
Better	26.2 / 27.0 (17)	15.4 / 27.8 (10)	1.5 / 5.9 (1)	26.2 / 26.6 (17)	6.2 / 18.2 (4)	24.6 / 22.2 (16)	100 (65)	
Same	26.7 / 73.0 (46)	14.5 / 69.4 (25)	5.8 / 58.8 (10)	23.8 / 64.1 (41)	5.8 / 45.5 (10)	23.3 / 55.6 (40)	100 (172)	$\mathbf{\chi^2(10) =}$
Worse	0 / 0 (0)	2.7 / 2.8 (1)	16.2 / 35.3 (6)	16.2 / 9.4 (6)	21.6 / 36.4 (8)	43.2 / 22.2 (16)	100 (37)	**37.326,**

								**p < .001**
Total	23.0 / 100 (63)	13.1 / 100 (36)	6.2 / 100 (17)	23.4 / 100 (64)	8.0 / 100 (22)	26.3 / 100 (72)	100 (274)	
**Change in involvement in social groups during COVID-19**								
Less	22.3 / 72.9 (43)	8.3 / 47.1 (16)	6.7 / 92.9 (13)	26.4 / 85.0 (51)	9.3 / 85.7 (18)	26.9 / 81.3 (52)	100 (193)	**χ2(10) =**
Same	26.5 / 22.0 (13)	32.7 / 47.1 (16)	0 / 0 (0)	14.3 / 11.7 (7)	6.1 / 14.3 (3)	20.4 / 15.6 (10)	100 (49)	**26.183**
More	30.0 / 5.1 (3)	20.0 / 5.9 (2)	10.0 / 7.1 (1)	20.0 / 3.3 (2)	0 / 0 (0)	20.0 / 3.1 (2)	100 (10)	**p = .004**
Total	23.4 / 100 (59)	13.5 / 100 (34)	5.6 / 100 (14)	23.8 / 100 (60)	8.3 / 100 (21)	25.4 / 100 (64)	100 (252)	

statistically significant (p<.05) **in bold**

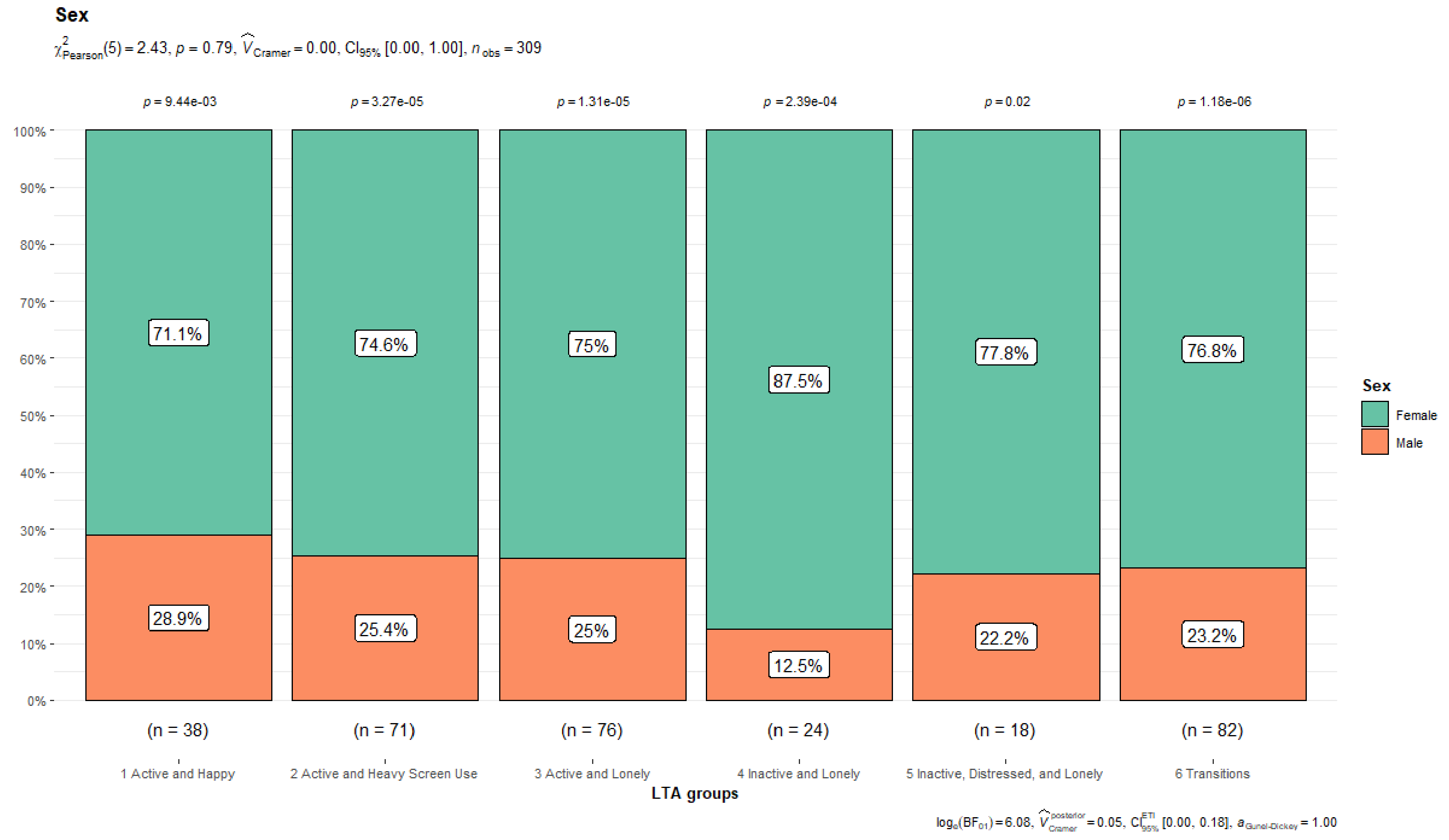

Figure S3 LTA group comparison by Sex (ns.)

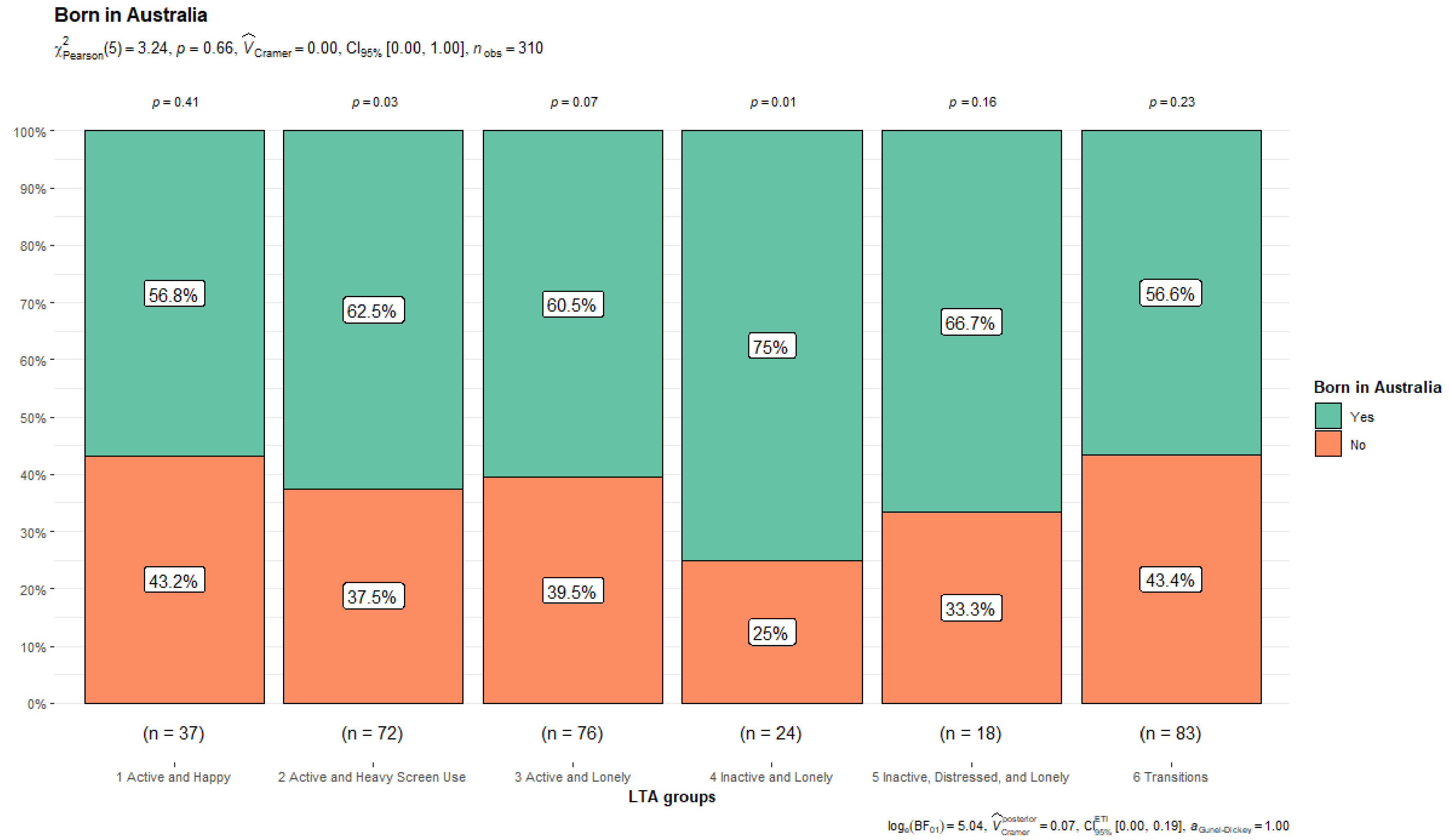

Figure S4 LTA group comparison by Country of Origin (ns.)

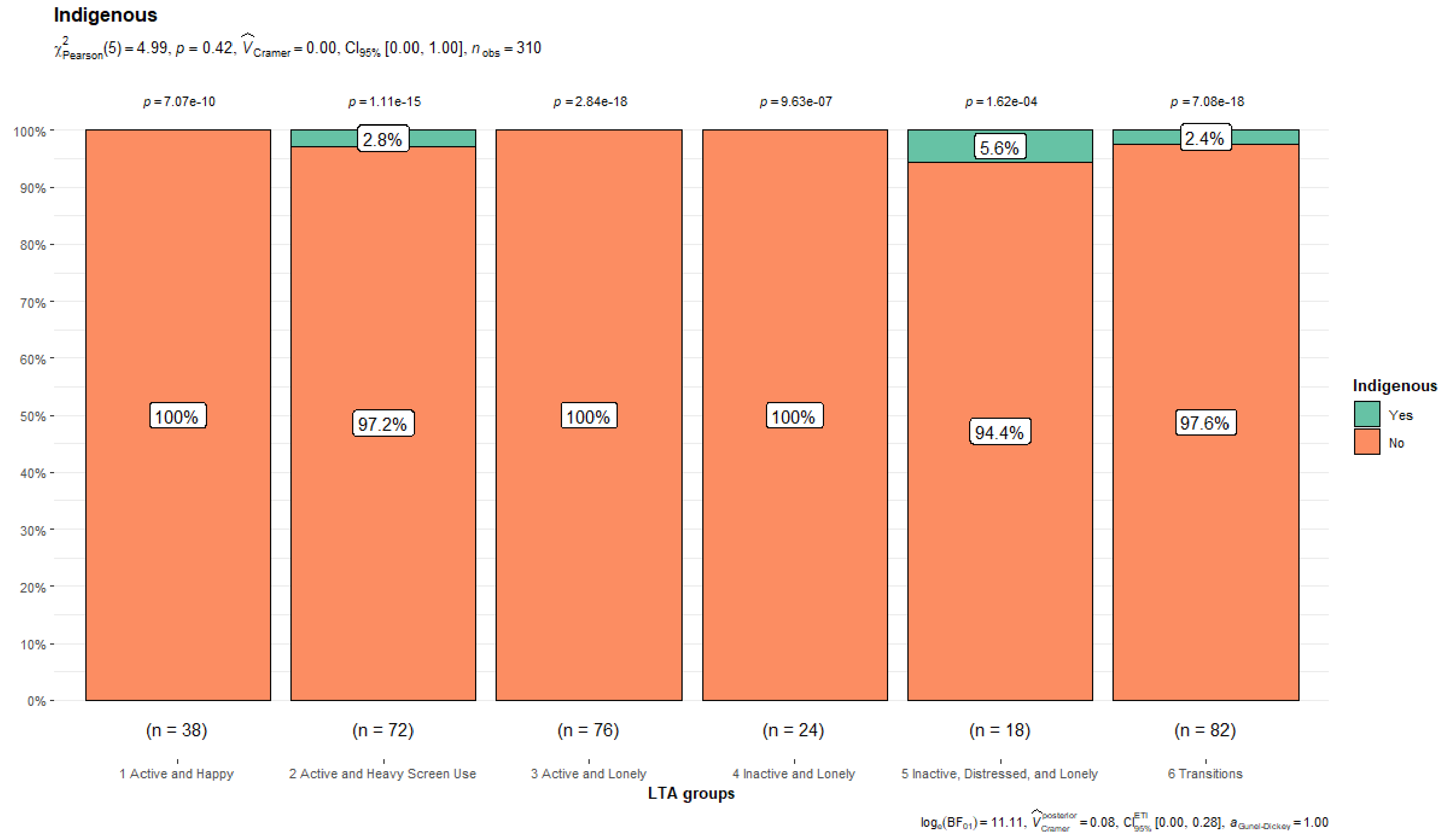

Figure S5 LTA group comparison by Indigenous background (ns.)

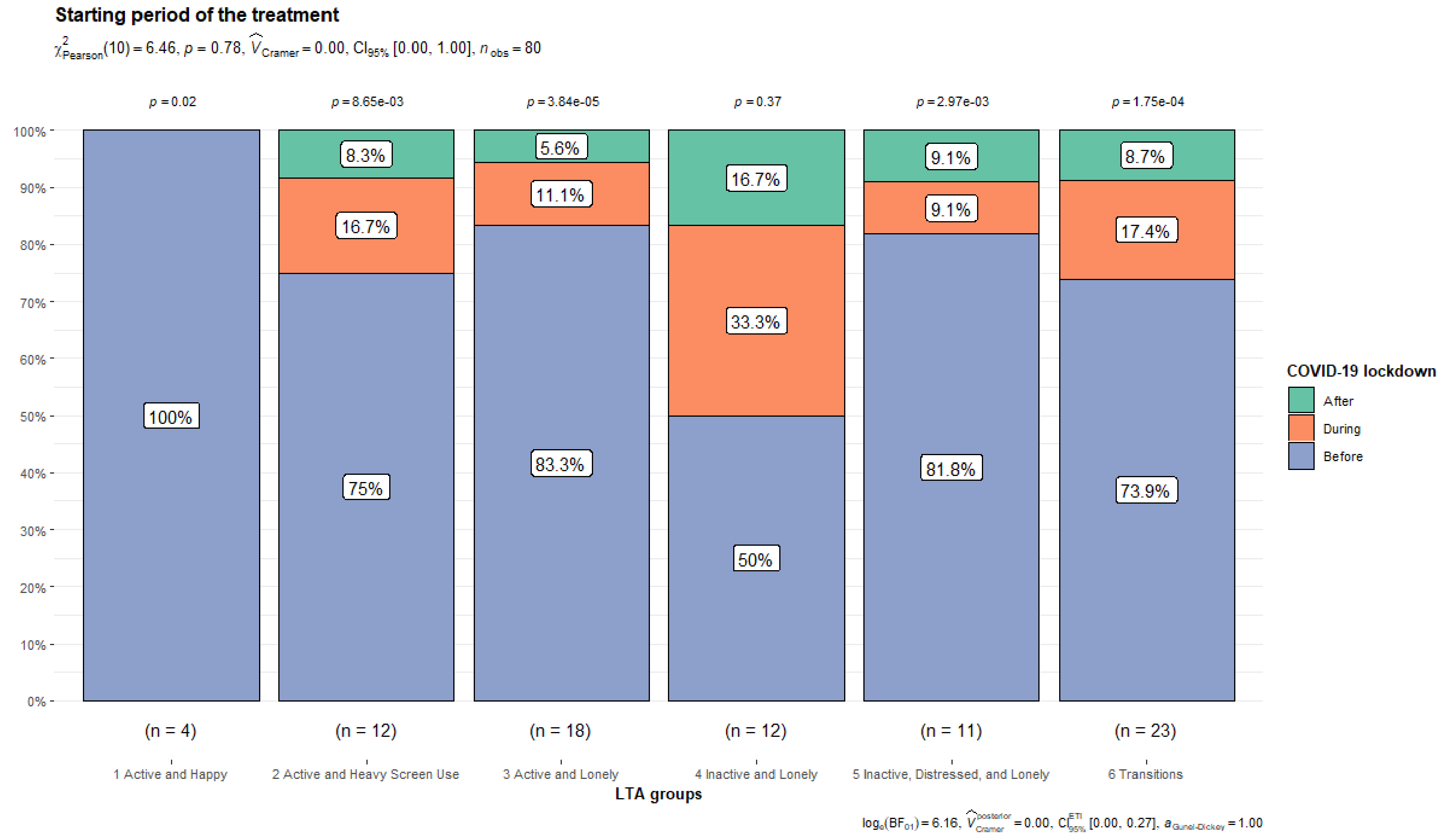

Figure S6 LTA group comparison by Starting period of the treatment (ns.)

Table S11 Multiple responses (Open-ended question): Effects of COVID-19

Multiple responses – OE questions	Active and heavy screen use (n = 72)	Active and happy (n = 38)	Inactive, distressed and lonely (n = 18)	Active and lonely (n = 76)	Inactive and lonely (n = 24)	Transition (n = 85)
**Are there any other comments you would like to make about the impact of COVID-19 on physical and mental well-being?**	n = 42	n = 26	n = 11	n = 42	n = 16	n = 45
None	16.7 (7)	34.6 (9)	9.1 (1)	11.9 (5)	12.5 (2)	33.3 (15)
Governmental & media -related commentary	26.2 (11)	26.9 (7)	27.3 (3)	14.3 (6)	18.8 (3)	22.2 (10)
Mention of the risk groups	9.5 (4)	.	.	2.4 (1)	.	2.2 (1)
Positive impacts of COVID-19	40.5 (17)	57.7 (15)	45.5 (5)	47.6 (20)	25.0 (4)	24.4 (11)
Mental health issues, concerns, fears, etc.,	35.7 (15)	15.4 (4)	63.6 (7)	33.3 (14)	43.8 (7)	28.9 (13)
Social relations (unable to see, etc.)	2.4 (1)	3.8 (1)	.	7.1 (3)	12.5 (2)	11.1 (5)
Other	4.8 (2)	.	.	.	6.3 (1)	4.4 (2)
Total % (n)	135.7 (57)	138.5 (36)	145.5 (16)	116.7 (49)	118.8 (19)	126.7 (57)
**Provide examples of how you and your family were positively affected**	n = 54	n = 27	n = 8	n = 50	n = 15	n = 53
Social relations						
More family activity	37.0 (20)	59.3 (16)	50.0 (4)	42.0 (21)	33.3 (5)	60.4 (32)
Zoom/phone with family and/or friends living elsewhere	18.5 (10)	14.8 (4)	37.5 (3)	10.0 (5)	26.7 (4)	11.3 (6)
Improved communications	14.8 (8)	14.8 (4)	.	18.0 (9)	13.3 (2)	18.9 (10)
Work-life balance						
Fewer social/work engagements	9.3 (5)	22.2 (6)	12.5 (1)	22.0 (11)	13.3 (2)	7.5 (4)
More time to do the things I wanted	35.2 (19)	18.5 (5)	25.0 (2)	26.0 (13)	6.7 (1)	20.8 (11)
More time to exercise or be active	20.4 (11)	14.8 (4)	12.5 (1)	20.0 (10)	6.7 (1)	3.8 (2)
More time cooking	5.6 (3)	.	12.5 (1)	2.0 (1)	6.7 (1)	1.9 (1)
Saved some money	16.7 (9)	18.5 (5)	37.5 (3)	6.0 (3)	6.7 (1)	17.0 (9)
Self-reflection						
More peaceful	18.5 (10)	11.1 (3)	.	22.0 (11)	20.0 (3)	18.9 (10)
Space to re-evaluate life/ reflect	9.3 (5)	7.4 (2)	12.5 (1)	14.0 (7)	13.3 (2)	11.3 (6)
Total % (n)	185.2 (100)	181.5 (49)	200.0 (16)	182.0 (91)	146.7 (22)	171.7 (91)
**Provide examples of how you and your family were negatively affected**	n = 56	n = 25	n = 12	n = 51	n = 20	n = 55
Social relations						
Couldn’t catch up with family and friends	62.5 (35)	32.0 (8)	41.7 (5)	43.1 (22)	75.0 (15)	56.4 (31)

Concerned for family members	8.9 (5)	8.0 (2)	.	7.8 (4)	.	18.2 (10)
Couldn’t attend important family events	7.1 (4)	16.0 (4)	.	7.8 (4)	10.0 (2)	3.6 (2)
Forced to spend time together	19.6 (11)	16.0 (4)	33.3 (4)	3.9 (2)	5.0 (1)	5.5 (3)
Limitations in leisure time						
Couldn’t exercise as pool/gym was closed	1.8 (1)	.	.	5.9 (3)	5.0 (1)	7.3 (4)
Couldn’t travel	32.1 (18)	32.0 (8)	33.3 (4)	21.6 (11)	15.0 (3)	16.4 (9)
Work-life balance						
Difficult working from home, working more or lost job	8.9 (5)	24.0 (6)	16.7 (2)	23.5 (12)	5.0 (1)	20.0 (11)
Health concerns						
Increased anxiety and fear	23.2 (13)	24.0 (6)	33.3 (4)	27.5 (14)	40.0 (8)	36.4 (20)
Loss of basic freedoms	10.7 (6)	12.0 (3)	33.3 (4)	9.8 (5)	15.0 (3)	3.6 (2)
Difficulties accessing healthcare	.	.	8.3 (1)	2.0 (1)	.	.
Drank too much	1.8 (1)	.	.	.	.	3.6 (2)
Total % (n)	176.8 (99)	164.0 (41)	200.0 (24)	152.9 (78)	170.0 (34)	170.9 (94)

Table S12 Multiple responses (Open-ended question): Health promotion campaigns

Multiple responses – OE questions	Active and heavy screen use (n = 72)	Active and happy (n = 38)	Inactive, distressed and lonely (n = 18)	Active and lonely (n = 76)	Inactive and lonely (n = 24)	Transition (n = 85)
**Please list the topic of any of the health promotion campaigns you recall that run during the COVID-19 lockdown period.**	n = 57	n = 29	n = 11	n = 52	n = 21	n = 60
None	21.1 (12)	27.6 (8)	45.5 (5)	11.5 (6)	14.3 (3)	33.3 (20)
Hygiene						
Washing hands	54.4 (31)	48.3 (14)	36.4 (4)	61.5 (32)	71.4 (15)	38.3 (23)
Coughing into elbow	10.5 (6)	3.4 (1)	9.1 (1)	9.6 (5)	28.6 (6)	5.0 (3)
Flu vaccination	1.8 (1)	.	.	7.7 (4)	9.5 (2)	3.3 (2)
Masks	5.3 (3)	10.3 (3)	.	1.9 (1)	.	3.3 (2)
Regulations						
Social distancing	47.7 (27)	48.3 (14)	18.2 (2)	36.5 (19)	47.6 (10)	31.7 (19)
Stay home	7.0 (4)	.	.	3.8 (2)	4.8 (1)	6.7 (4)
CovidSafe app	5.3 (3)	3.4 (1)	.	5.8 (3)	4.8 (1)	5.0 (3)
Mental and social health						
Mental health	14.0 (8)	3.4 (1)	9.1 (1)	19.2 (10)	19.0 (4)	13.3 (8)
Stay connected to one another	8.8 (5)	10.3 (3)	9.1 (1)	5.8 (3)	.	3.3 (2)
Domestic violence awareness	3.5 (2)	.	.	.	.	.
Physical health						
Physical activity	3.5 (2)	.	.	7.7 (4)	4.8 (1)	8.3 (5)
Reduce alcohol	3.5 (2)	3.4 (1)	.	5.8 (3)	4.8 (1)	5.0 (3)
Other	3.5 (2)	6.9 (2)	.	11.5 (6)	9.5 (2)	5.0 (3)
Total % (n)	189.5 (108)	165.5 (48)	127.3 (14)	188.5 (98)	219.0 (46)	161.7 (97)
**Please describe any changes you made to your behaviours as a result of these campaigns**	n = 53	n = 26	n = 10	n = 53	n = 20	n = 58
No change	41.5 (22)	42.3 (11)	60.0 (6)	24.5 (13)	40.0 (8)	41.4 (24)
Hygiene						
Washed hand properly	32.1 (17)	34.6 (9)	30.0 (3)	45.3 (24)	30.0 (6)	25.9 (15)
Used sanitizer	15.1 (8)	23.1 (6)	.	34.0 (18)	15.0 (3)	8.6 (5)
Coughed into elbow	9.4 (5)	3.8 (1)	10.0 (1)	5.7 (3)	.	1.7 (1)
Avoided touching my face	1.9 (1)	7.7 (2)	.	7.5 (4)	.	1.7 (1)
Reduced touching surfaces when out	3.8 (2)	.	.	5.7 (3)	.	.
Disinfected surfaces	5.7 (3)	3.8 (1)	.	5.7 (3)	.	3.4 (2)
Regulations						

Avoided socialising	5.7 (3)	3.8 (1)	.	3.8 (2)	5.0 (1)	3.4 (2)
Social distanced	39.6 (21)	34.6 (9)	10.0 (1)	39.6 (21)	45.0 (9)	.
Stayed home more	9.4 (5)	.	10.0 (1)	.	5.0 (1)	20.7 (12)
Mental and social health						
Stopped listening to the news	.	.	10.0 (1)	1.9 (1)	.	5.2 (3)
Tried to stay in touch with people	1.9 (1)	.	.	3.8 (2)	5.0 (1)	6.9 (4)
Other	5.7 (3)	.	10.0 (1)	13.2 (7)	15.0 (3)	3.4 (2)
Total % (n)	171.7 (91)	153.8 (40)	140.0 (14)	109.6 (101)	160 (32)	129.3 (75)
**What health promotion messages should have been provided to the community during COVID-19 lockdown that were missing at the time?**	n = 53	n = 27	n = 8	n = 53	n = 16	n = 53
None	24.5 (13)	29.6 (8)	25.0 (2)	32.1 (17)	50.0 (8)	30.2 (16)
Hygiene						
Better cleaning information	3.8 (2)	3.7 (1)	.	.	.	1.9 (1)
Clearer direction as to when and where to wear masks	17.0 (9)	14.8 (4)	.	15.1 (8)	12.5 (2)	7.5 (4)
More details about testing procedure	1.9 (1)	.	.	1.9 (1)		1.9 (1)
Regulations						
Better access to timely WA specific information via web	15.1 (8)	18.5 (5)	25.0 (2)	26.4 (14)	25.0 (4)	17.0 (9)
Better self-isolation information	1.9 (1)	.	.	1.9 (1)	.	1.9 (1)
Better social distancing options	.	.	12.5 (1)	1.9 (1)	6.3 (1)	1.9 (1)
More clarity when to stay home	.	3.7 (1)	.	.	.	1.9 (1)
Stricter inforcement	3.8 (2)	.	.	3.8 (2)	6.3 (1)	.
Mental, physical, and social health						
More about physical, nutritional and mental well-being	13.2 (7)	14.8 (4)	25.0 (2)	15.1 (8)	.	20.8 (11)
More details on how and where to access (mental) healthcare	3.8 (2)	.	.	1.9 (1)	6.3 (1)	5.7 (3)
Strategies for coping	1.9 (1)	11.1 (3)	.	.	6.3 (1)	1.9 (1)
Information on how to better stay in touch with people	1.9 (1)	3.7 (1)	.	7.5 (4)	.	3.8 (2)
Domestic violence	.	.	12.5 (1)	.	.	1.9 (1)
Public health message						
More age and culturally specific advertisements	1.9 (1)	3.7 (1)	12.5 (1)	5.7 (3)	.	.
Emphasize the need of the community over the individual	11.3 (6)	.	.	3.8 (2)	.	5.7 (3)
Interviews with people who had the virus	.	.	.	.	.	3.8 (2)
Lockdown was a mistake	3.8 (2)	3.7 (1)	.	.	.	1.9 (1)
Other	7.5 (4)	14.8 (4)		1.9 (1)	6.3 (1)	3.8 (2)
Total % (n)	113.2 (60)	122.2 (33)	112.5 (9)	118.9 (63)	118.8 (19)	113.2 (60)
